# Modeling the Probability of HIV Infection over Time in High-Risk Seronegative Participants Receiving Placebo in Five Randomized Double-Blind Placebo-Controlled HIV Pre-Exposure Prophylaxis Trials: A Patient-Level Pooled Analysis

**DOI:** 10.3390/pharmaceutics14091801

**Published:** 2022-08-27

**Authors:** Maria Garcia-Cremades, Craig W. Hendrix, Priya Jayachandran, Natasha Strydom, Leah Jarlsberg, Robert Grant, Connie L. Celum, Michael Martin, Jared M. Baeten, Jeanne Marrazzo, Peter Anderson, Kachit Choopanya, Suphak Vanichseni, David V. Glidden, Radojka M. Savic

**Affiliations:** 1 Department of Bioengineering and Therapeutic Sciences, University of California San Francisco, San Francisco, CA 94143, USA; 2 Division of Clinical Pharmacology, Department of Medicine, Johns Hopkins University, Baltimore, MD 21287, USA; 3 Department of Medicine, University of California San Francisco, San Francisco, CA 94143, USA; 4 Departments of Global Health, Medicine, and Epidemiology, University of Washington, Seattle, WA 98104, USA; 5 Division of HIV/AIDS Prevention, Centers for Disease Control and Prevention, National Center for HIV/AIDS, Viral Hepatitis, STD, and TB Prevention, Atlanta, GA 30333, USA; 6 Thailand Ministry of Public Health—US CDC Collaboration, Nonthaburi 11000, Thailand; 7 Division of Infectious Diseases, University of Alabama at Birmingham Medical Center, Birmingham, AL 35294, USA; 8 Department of Pharmaceutical Sciences, University of Colorado, Denver, CO 80045, USA; 9 Bangkok Tenofovir Study Group, Bangkok 10600, Thailand; 10 Department of Epidemiology and Biostatistics, University of California San Francisco, San Francisco, CA 94143, USA

**Keywords:** HIV prevention trials, key and vulnerable populations, risk factors, risk phenotypes, modeling

## Abstract

The World Health Organization recommends pre-exposure prophylaxis (PrEP) for individuals at substantial risk of HIV infection. The aim of this analysis is to quantify the individual risk of HIV infection over time, using a large database of high-risk individuals (*n* = 5583). We used data from placebo recipients in five phase III PrEP trials: iPrEx, conducted in men who have sex with men and transgender women; VOICE, conducted in young women at high sexual risk; Partners PrEP, conducted in HIV serodiscordant heterosexual couples; TDF2, conducted in high-risk heterosexual men and women; and BTS, conducted in persons who inject drugs. The probability of HIV infection over time was estimated using NONMEM7.4. We identified predictors of HIV risk and found a substantial difference in the risk of infection among and within trial populations, with each study including a mix of low, moderate, and high-risk individuals (*p* < 0.05). Persons who were female at birth were at a higher risk of HIV infection than people who were male at birth. Final models were integrated in a tool that can assess person-specific risk and simulate cumulative HIV risk over time. These models can be used to optimize future PrEP clinical trials by identifying potential participants at highest risk.

## 1. Introduction

In 2020, the World Health Organization (WHO) estimated that 37.7 million people were living with human immunodeficiency virus (HIV), 1.5 million had newly acquired HIV, and 608,000 people with HIV had died [1]. While new infections and HIV-related deaths have considerably declined between 2000 and 2020, the HIV pandemic continues to be a major contributor to the global burden of disease [1].

Antiretroviral therapy (ART) reduces the risk of HIV infection when administered as pre-exposure prophylaxis (PrEP) and post-exposure prophylaxis (PEP) [1]. The effectiveness of PrEP at reducing HIV transmission has been demonstrated in several randomized controlled trials (RCTs) [2,3,4,5,6,7,8,9,10], and its use has been increasing globally, with more than 600,000 people in 76 countries worldwide having received oral PrEP at least once in 2019 [11]. However, there is still considerable room for improvement in the widespread use of PrEP in those at high risk of infection, and even in settings where access to and use of PrEP is high, adherence is a challenge and varies among different populations [12].

Defining the risk of HIV infection remains a challenge and limits efforts to direct HIV prevention services, including PrEP, to people who would benefit most. The WHO recommends PrEP to populations with a substantial risk for acquiring HIV, which is defined as an HIV incidence > 3 per 100 person-years. However, HIV risk varies within populations based on individual characteristics and behaviors [13]. If the characteristics of high-risk individuals and predictors of HIV infection within key populations can be identified, PrEP provision could be better directed to populations who would benefit most, thereby reducing HIV transmission. Likewise, the design of future PrEP clinical trials could be optimized by selecting individuals at highest risk of HIV infection with greater anticipated incidence.

Previous studies used data from several PrEP randomized controlled trials (RCTs) to identify predictors of HIV infection and develop heterogeneous risk scoring tools in specific populations such as men who have sex with men (MSM), heterosexual serodiscordant couples, and heterosexual women [14,15,16,17].

In this study, we aimed to characterize and compare the probability of HIV infection within high-risk populations using data from the placebo arms of five PrEP clinical trials: the Pre-exposure Prophylaxis Initiative (iPrEx) trial [2], the Vaginal and Oral Interventions to Control the Epidemic (VOICE) trial [3], Partners Pre-exposure Prophylaxis (Partners PrEP) trial [4], the Bangkok Tenofovir Study (BTS) [5] and the Botswana TDF/FTC Oral HIV Prophylaxis Trial (TDF2) trial [6]. By quantifying the risk factors within each population, we attempted to identify subgroups at low, medium, and high risk of acquiring HIV in each population. With this quantitative understanding of HIV risk in key populations based on individual characteristics and risk behaviors, we may be able to predict HIV incidence in the absence of intervention and optimize future HIV prevention clinical trials by enrolling the individuals at highest risk.

## 2. Materials and Methods

### 2.1. Database: Clinical Trials and Study Design

We included participants enrolled in the placebo arms of five phase III clinical trials that evaluated the efficacy of daily TDF, administered with or without FTC, as PrEP in populations at high risk of HIV infection. These RCTs were conducted, between 2005 and 2013, among: MSM/transgender women (TGW) (iPrEx, NCT00458393) [2], young heterosexual women at high sexual risk (VOICE, NCT00705679) [3], heterosexual men and women in HIV serodiscordant partnerships (Partners PrEP, NCT00557245) [4], people who inject drugs (Bangkok Tenofovir Study [BTS], NCT00119106) [5], and heterosexual men and women at high risk (TDF2, NCT00448669) [6]. Trial protocols were reviewed and approved by local and institutional Ethical Committees, and all participants provided written informed consent. Trials included in this analysis were double-blind, with neither the participants nor the researcher knowing participants were receiving placebo or active agent. The randomization process was specific for each trial and can be found in the trial primary publications [2,3,4,5,6]. Overall, in all the trials, study visits were scheduled every month after enrollment, which included drug dispensation, adherence counseling and monitoring, medical history, and HIV status tests. For this study, we included data from trial participants who were HIV negative at enrollment and had the date of enrollment and the date of HIV infection or the date their data were censored in the trial database. We collected longitudinal HIV test results (collected monthly), and baseline demographic characteristics, sexual transmitted infections (STIs), and risk behavior (Table 1).

### 2.2. Data Analysis

Primary analysis consisted of characterizing the probability of acquiring HIV infection over time of initially seronegative individuals and identifying predictors of infection through parametric survival analysis [18] using NONMEM7.4 [19] with the LAPLACE estimation method. This probability was treated as a right-hand censored time to event variable. The primary outcome was time to HIV seroconversion of initially seronegative individuals, which is defined as the time from the date of enrollment to the date of seroconversion and characterized using a parametric time to event model. These models are characterized by a hazard (hz) that can be parameterized using different distribution functions. The link between the hz and the overall survival probability (OS) (defined as the probability of remaining HIV uninfected) is established through the cumulative hazard (HZ) over time (OS = e-HZ) [18]. The identification of the different predictors of HIV infection was performed by covariate analysis in the hz baseline parameters. The covariates included in the analysis were the population characteristics and risk factors of participants collected at study baseline. Hazard ratios (HR) were computed for each covariate included in the final multivariate model to compare the effect magnitude of each predictor on the relative risk. The HR was computed by calculating the ratio of the baseline hazard of group A (e.g., No use of condom) to group B (e.g., Use of condom). For continuous predictors, HR was calculated with respect to the median value of the population (e.g., being 20 years old increases the risk of contracting HIV with respect to being 24 years old in the iPrEx study). Internal model evaluation was performed by simulation-based diagnostics using Kaplan–Meier Visual Predictive Check (VPC) plots [20,21] stratified by significant predictors. Detailed explanation of model building and evaluation is included in the Supplementary Methods. Exploratory analysis by Kaplan–Meier plots stratified by the different covariates and univariate Cox-regression analysis was performed to identify preliminary potential predictors of HIV infection and to support the final model.

### 2.3. Risk Stratification Using Final Multivariate Model

Risk stratification was performed based on a target probability of remaining HIV uninfected at 1 year of follow up (52 weeks). We defined low risk as <1% probability of HIV infection, moderate risk as 1% to 5% probability, and high risk as >5% probability. A risk score (RS) corresponding to these probabilities was calculated by integrating the survival function (RS1% = −(log(OS99%)/52)). Individual risk scores were computed based on the significant predictors using the baseline hazard for the final model describing time to HIV infection. These computed risk scores were used to distribute individuals into the risk subgroups (low, moderate, high).

### 2.4. Interactive Web Application

The final models were coded using the mlxR package (https://cran.r-project.org/package=mlxR, accessed on 1 July 2019) and were incorporated into an interactive web application using the R package shiny (https://shiny.rstudio.com, accessed on 1 July 2019) to perform interactive simulations of individual HIV risk profiles within each key population based on individual predictors.

## 3. Results

### 3.1. Largest Dataset Regarding HIV Outcome including Different High-Risk Populations

From the placebo arms of the five PrEP phase III clinical trials, 5583 individuals were included in the analysis: 1218 MSM/TGW (iPrEx), 2008 young women at high risk (VOICE), 1574 HIV negative partners in HIV-serodiscordant couples (Partners PrEP), 177 people who inject drugs (BTS), and 606 heterosexual men and women at high risk (TDF2). A detailed description of the baseline characteristics and population-specific risk behaviors collected in the five trials is shown in Supplementary Table S1a–e. Due to the different risk factors and demographic information collected in each study (Table 1 and Table S1a–e), and the large number of individuals enrolled, each study population was analyzed separately. The Supplementary Materials (Preliminary analysis) show the results from the preliminary Kaplan–Meier plot (Supplementary Figures S1–S5) and univariate cox-regression analysis (Supplementary Tables S2–S6) for each study population.

### 3.2. Substantial Differences in Risk Infection between Different Key Populations

**Figure 1:**
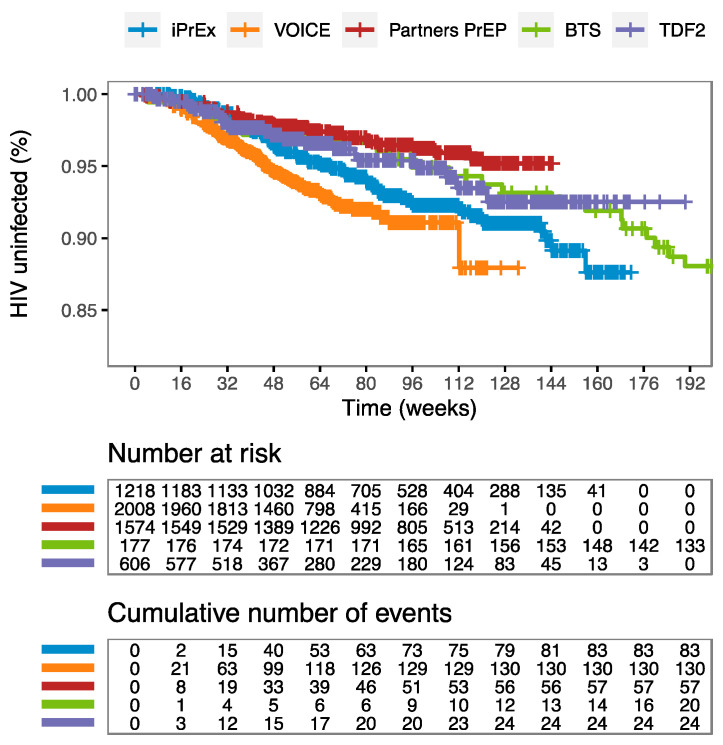
Probability of HIV infection over time of the placebo arms of the five PrEP studies. The Bangkok study’s follow up was until week 348. Vertical lines represent the censored individuals. Number at risk represents the number of individuals at HIV risk in each study at each time of the *x*-axis. Cumulative number of events represents the number of individuals who contracted HIV infection in each study at each time of the *x*-axis.

The risk of acquiring HIV over time varied among the studies (Figure 1). Women at high risk (VOICE) showed the highest risk of HIV infection (HIV uninfected < 90% at week 130); and serodiscordant heterosexual partners (Partners PrEP) showed the lowest risk of HIV infection (HIV uninfected ≈ 95% at week 130). The probability of remaining HIV uninfected over time was characterized by a parametric survival model using an exponential distribution in which the hz over time is equal to a baseline hazard parameter (λ) (hz(t) = λ). Differences in study populations were considered by estimating a different λ value for each population at high risk (Table 2). The hazard function was modified by including significant predictors of HIV risk as covariates of λ using a linear relationship. Predictor estimates and their effect on HIV risk are summarized in Table 2.

### 3.3. Low, Moderate, and High-Risk Profiles Inside Key Populations Based on the Significant Predictors

**Figure 2:**
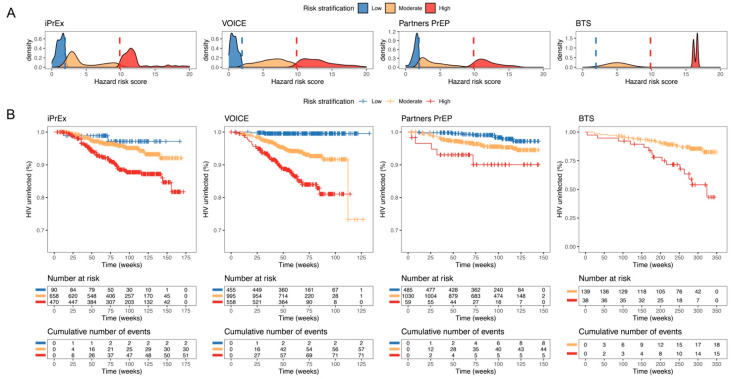
Risk stratification representation. (**A**) Distribution of individual risk scores and (**B**) Observed Kaplan–Meier rates stratified by low, medium, and high-risk group for each study population. Low, medium, and high-risk groups were defined as individuals with a probability of infection over 1 year of <1%, between 1 and 5%, and >5%. No risk stratification is shown for TDF2 study as no available predictor was included in the model.

An RS of 1.93 × 10^−4^ was associated with a probability of HIV infection of 1% (low risk) and an RS of 9.86 × 10^−4^ was associated with a probability of 5% (high risk) at year 1 of follow-up. The distribution of individual RSs based on the predictors for each population is shown in Figure 2A.

A subgroup of individuals at low risk (RS ≤ 1.93 × 10^−4^) were identified constituting 8% (MSM/TGW), 23% (young women at high risk) and 31% (HIV negative partners in HIV-serodiscordant heterosexual couples) of their study population. Using this stratification, the population of people who inject drugs in the BTS study did not include low-risk individuals. High-risk individuals (RS ≥ 9.86 × 10^−4^) constituted 36% (MSM/TGW), 28% (young women at high risk), 4% (HIV negative partners in HIV-serodiscordant heterosexual couples) and 21% (people who inject drugs) of their study populations. These high-risk subpopulations show differences across the study populations, with a cumulative probability of acquiring HIV infection in 1 year of 6% (MSM/TGW), 11% (young women at high risk), 7% (HIV negative partners in HIV-serodiscordant heterosexual couples) and 8% (BTS). Moderate risk individuals (1.93 × 10^−4^ < RS > 9.86 × 10^−4^) represented the largest subgroup with individuals constituting 56% (MSM/TGW), 49% (young women at high risk), 65% (HIV negative partners in HIV-serodiscordant heterosexual couples), and 79% (people who inject drugs) of their study populations. We did not find statistically significant predictors of HIV infection in the TDF2 study, so the risk stratification was not applied to that data. The risk stratification based on the individual predictors shows a statistically significant difference in the risk of acquiring HIV infection within and across populations (Figure 2B).

Table 3 shows the distribution of participant characteristics in each risk stratum for each study population. Overall, individuals with high-risk characteristics are most prevalent in the high-risk stratum, in contrast to those with low-risk characteristics (Supplementary Table S7). Interestingly, all the individuals in the high-risk stratum in the Partners PrEP study are women.

**Figure 3:**
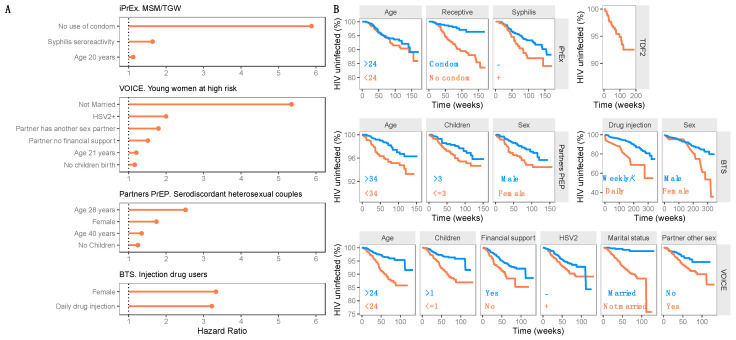
Model predictors and evaluation. (**A**) Effect magnitude of each predictor on the relative risk (hazard ratio) of HIV infection for each population. (**B**) VPCs by studies and by covariates as a Kaplan–Meier plot of HIV probability. Lines represent raw data, and shaded areas cover the 95% prediction interval calculated from 200 simulated studies. No predictor effect is represented for TDF2 study, as no available predictor was included in the model.

A comparison of each predictor effect on the HR for each population is shown in Figure 3A. A detailed description of model predictors in each study is included in Supplementary Material (Additional Results). The evaluation of the established models supports the adequacy of the parametric multivariate models and demonstrates good agreement between observed and simulated HIV risk data for each of the identified predictors (Figure 3B). The model evaluation plots support the following statements: (i) there are different levels of HIV risk in each study population (e.g., married women show a lower probability of HIV infection compared to unmarried women in the VOICE trial), and (ii) there is a difference in the high-risk profiles between the study populations (e.g., red profiles of Figure 3B for number of children predictors for VOICE vs. Partners PrEP studies).

### 3.4. Sex at Birth Effect: Females Appear to Be at Higher Risk of HIV Infection

In the trials that enrolled both men and women, women were at a higher risk of HIV infection. Being female in the Partners PrEP Study increased the relative hazard ratio by 1.74 points, and being female in the BTS increased the relative hazard ratio by 3.33 points. In TDF2, although no predictors were included in the final model, the Kaplan–Meier plot of the raw data shows women at higher risk of HIV infection (Supplementary Figure S5).

### 3.5. Tool to Estimate Risk of HIV Infection

We developed an online interactive web application to simulate how the risk profiles change for each study population depending on individual predictors. The web app is hosted at http://saviclab.org/hiv-risk/. The tool allows users to compare the probability of HIV infection over time for individuals within and between the key and to determinate the level of risk (low, medium, and high) for each simulated profile. The definition of low, medium, and high risk can be set in the tool by the user by selecting the risk limits (probability of HIV infection) for low and high risk at a given time of follow up. Supplementary Figure S6 provides a snapshot of the tool comparing the probability of HIV between populations based on the individual predictors.

## 4. Discussion

We characterized the probability of HIV infection of five study populations by integrating longitudinal data from the placebo arms of five pivotal PrEP RCTs. The parametric models identified statistically significant predictors of HIV infection in four of the five studies, allowing us to quantify the risk of HIV infection in high-risk individuals not receiving PrEP. We found a substantial difference in the risk of HIV infection among and within the trial populations, with each study population including a mix of low, moderate, and high-risk individuals. Females at birth are associated with a higher risk of infection in populations that included both sexes. Notably, this analysis constitutes the first comparison of substantially different HIV risk levels across key populations.

We developed a parametric multivariate model for each study population due to the heterogeneity of the risk factors across the studies. However, we initially attempted to develop a single parametric multivariate model to quantify the probability of HIV infection in the pooled placebo data from the five studies. This preliminary meta-analysis that included the study populations and their common available covariates (i.e., age, education, ethnicity, sex and high-risk population) identified age, sex and study population as significant predictors of HIV infection (*p* < 0.001). This approach, however, ignored other characteristics defined as high risk (e.g., prisoners) [22]. This analysis also failed to identify the population-specific predictors (e.g., receptive partner condom use among MSM/TGW, or drug injection frequency in people who inject drugs).

Female participants had a higher risk of infection in all studies that included women. While the underlying mechanism for this finding cannot be revealed from this modeling analysis, this result may be influenced by gender inequalities in the populations of women included in this analysis. These gender inequalities could be due to unequal distribution of HIV risk factors (e.g., difference in financial security, sexual behaviors, etc.), as well as the different effect of these risk factors (e.g., male-to-female transmission, concomitant sexually transmitted diseases, etc.) [23]. This finding is consistent with evidence that women account for more than half of the 36.9 million people living with HIV worldwide (African women comprise the largest component), and that three of five new HIV infections among young people (15–24 years) occur in females [24,25]. Unmarried women in the studies were at a higher risk of HIV infection than married women in the VOICE and Partners PrEP studies. Older age and having had more children lowered the risk of HIV infection by 7% per year increase in age and by 16% per child born to women in the VOICE study and by 25% per year increase in age to 34 years old and by 8% per child born to women in the Partners PrEP study. There is an urgent need to focus HIV prevention on young women at high sexual risk [26], especially in high HIV burden settings.

Our findings are consistent with previous studies that used risk assessment tools to predict HIV infection risk [14,16,17,27,28,29,30,31]. Supplementary Materials (Additional Discussion) includes a comparison of our findings with these studies. These risk assessment tools are largely based on descriptive and semi-parametric Cox-proportional hazard analyses, which are commonly used to quantify risk and identify predictors but cannot be used to simulate HIV risk over time based on individual’s characteristics. Our analysis was performed using parametric survival models in which the survival and hazard function are characterized by a distribution expressed in terms of parameters that can be estimated. The main advantages of the parametric survival models we used in this analysis are that, as the underlying hazard over time is fully characterized and estimated, simulations of the cumulative probability of HIV infection over time can be performed in different scenarios, for different individuals, based on the combination of the predictors included in the model (e.g., HIV risk for a 20-year-old man with a syphilis diagnosis, who has condomless receptive anal sex with men while not on PrEP). In addition, we integrated the models in a user-friendly interactive platform that overcomes the associated complexity of model computation. This tool integrates the models for predicting the probability of remaining HIV uninfected over time based on individual characteristics for the four study populations for which significant predictors were identified. It can help identify the combination of variables associated with the highest or lowest risk of infection compared to the median risk profile for a determinant population. Additionally, the tool can be updated by integrating information from ongoing and future PrEP RCTs to continue to refine and improve the estimates [32]. The integration of new data could help to refine and validate the current models, acquiring more precision in the parameters estimates, and it could help to identify new predictors of HIV risk in those population where this analysis was unable to find them (e.g., heterosexual men at high risk of infection, from TDF2 study). The tool serves as a companion interactive app for the manuscript, and it could be used to stimulate conversation about HIV prevention and as an informational tool for assisting on efficient future trial planning.

Our analysis had several limitations. The studies were conducted among specific populations and geographic locations, and the results may not be generalizable to other populations and sites. Unfortunately, this analysis was unable to find significant predictors of HIV risk in the TDF2 study, which includes data from a population of heterosexual men and women at high risk of infection. In addition, some participant characteristics (e.g., partner viral load (Partners PrEP) or drug use (iPrEx)) that have been found to be associated with HIV infection [14] were not included in our models because of missing data. For example, among the 1574 Partner PrEP participants, only 85 had partner viral load information available. All 49 participants whose partners had quantifiable viral load acquired HIV during follow-up, and none of the 36 participants whose partners had undetectable viral loads became infected. This is consistent with studies indicating essentially no HIV transmission risk when viral load is below detection limits [33]. In addition, the models were study-specific, so it was not possible to include the effect on HIV risk of the combination of characteristics across all of the different study populations. Lastly, the data used are slightly dated, with participants enrolled over ten years ago. Therefore, future plans include the collection of contemporary data and key populations to validate and refine this analysis.

## 5. Conclusions

In conclusion, the parametric models we used allowed us to identify populations at low, medium, and high risk of HIV infection. Our findings can be used to improve HIV prevention trial recruitment by focusing on individuals at highest risk of HIV infection with greater anticipated incidence. Furthermore, these models can be used to inform risk without bias in a population receiving PrEP as prevention therapy by differentiating between the protective effect of PrEP and the underlying HIV risk based on the identified predictors [34]. Future plans include adding new placebo arms from new clinical trials to the existing framework to promote knowledge integration and data sharing and to increase the predictive power of the analysis.

## Figures and Tables

**Table 1:** Population characteristics and risk factors of participants in five pre-exposure prophylaxis studies.

Study	Population	Geographical Region	Demographics	Sexually Transmitted Infections	Sexual Risk Behavior	Other Risk Factors
iPrEx	MSM/TGW	- Peru - Ecuador - South Africa - Brazil - Thailand - US	- Age - Education - Ethnicity - Sex at birth - Marital status - Transgender	- HSV2 - Gonorrhea - Chlamydia - Syphilis	- No. of partners - No. of intercourses - Protective intercourse - Receptive/insertive partner	- Partner’s HIV status
VOICE	Young women at high sexual risk	- South Africa - Uganda - Zimbabwe	- Age - Education - Ethnicity - Financial support (by partner) - Sex at birth - Marital status - N° live birth	- HSV1-2 - Gonorrhea - Chlamydia - Syphilis - Trichomonas	- No. of partners - No. of intercourses - Protective intercourse - Type of intercourse (vaginal/anal)	- Partner’s HIV status - Partner circumcised - Partner has other sexual partners
Partners PrEP	HIV negative partner in HIV- serodiscordant heterosexual couples	- Kenya - Uganda	- Age - Education - Sex at birth - Income - Marital status - N° Children - Pregnancy	- HSV2 - Gonorrhea - Chlamydia - Syphilis - Trichomonas	- No. of partners - No. of intercourses - Protective intercourse	- Partner’s viral load - Male circumcision
BTS	People who inject drugs	- Bangkok (Thailand)	- Age - Education - Ethnicity - Sex at birth		- No. of partners - No. of intercourses - Protective intercourse - Frequency anal intercourse	- Drug injection frequency - Live with - partner
TDF2	Heterosexual men/women at high risk	- Botswana	- Age - Education - Ethnicity - Sex at birth - Marital status	- Gonorrhea - Chlamydia - Syphilis - Trichomonas	- No. of partners - No. of intercourses - Protective intercourse	- HIV positive partner

Sexual risk behavior factors were recorded differently between studies (see Table S1a–e in Supplementary Materials).

**Table 2:** Final multivariate model by study.

Study	Model Building	Significance	↑ Risk	Parameters	Estimates (RSE%)
iPrEx	Baseline (Exponential)	-	-	$\theta_{\lambda }$	0.00103 (14%)
	+Predictor *Condom Receptive Partner*	*p* < 0.001	No use of condom when receptive partner	$\theta_{RECEPTIVE\prime }=1+\theta_{Receptive\;\left(\;\right)}$	$\theta_{Receptive\;\left(No\;condom\right)}\;$= 0 $\theta_{Receptive\;\left(Condom\right)}=-$0.73 (12%)
	+Predictor *Age*	*p* < 0.05	Younger age	$\theta_{AGE\prime }=1+\theta_{Age}\times \left(age-24\right)$	$\theta_{Age}=$−0.0305 (34%)
	+Predictor *Syphilis Seroreactivity*	Kaplan–Meier analysis	Syphilis positive	$\theta_{SYPHILIS\;\prime =1+\theta_{Syphilis\;\left(\;\right)}}$	$\theta_{Syphilis\left(-\right)}=0$
					$\theta_{Syphilis\left(+\right)}=$0.64 (70%)
VOICE	Baseline (Exponential)	-	-	$\theta_{\lambda }$	0.001 (15%)
	+Predictor *Herpes2*	*p* < 0.05	Herpes simplex virus 2 Positive	$\theta_{HSV2\prime }=1+\theta_{HSV2\;\left(\;\right)}$	$\theta_{HSV2\left(+\right)}\;$= 0.997 (37%) $\theta_{HSV2\left(-\right)}$ = 0
	+Predictor *Marital Status*	*p* < 0.001	Not being married	$\theta_{MARITAL\prime }=1+\theta_{Marital\;\left(\;\right)}$	$\theta_{Marital\left(Married\right)}$=−0.813 (12%) $\theta_{Marital\left(No\;Married\right)}$= 0
	+Predictor *Age*	*p* < 0.001	Younger age	$\theta_{AGE\prime }=1+\theta_{Age}\times \left(age-24\right)$	$\theta_{Age}=$−0.068 (26%)
	+Predictor *Primary Sex Partner has Sex with another Partner (last 3 months)*	*p* < 0.05	Primary sex partner has sex with another partner	$\theta_{PARTNER\prime \;}=1+\theta_{Partner\;\left(\;\right)}$	$\theta_{Partner\left(No\;extra\;sex\;\right)}=$−0.443 (32%) $\theta_{Partner\left(Extra\;sex\;\right)}=$0
	+Predictor *Financial Support*	*p* < 0.05	No financial support	$\theta_{FINANCIAL\;SUPPORT\prime }=1+\theta_{Support\left(\;\right)}$	$\theta_{Support\left(No\right)}$= 0.512 (60%) $\theta_{Support\left(Yes\right)}$= 0
	+Predictor *Number of Children Birth*	Kaplan Meier analysis	Lower number of children birth	$\theta_{CHILDREN\prime }=1+\theta_{Children}\times \left(children-1\right)$	$\theta_{Children}=$−0.162 (84%)
Partners PrEP	Baseline (Exponential)	-	-	$\theta_{\lambda }$	0.000166 (43%)
	+Predictor *Age*	*p* < 0.01	Younger age	IF age ≤34	$\theta_{Age^{1}}\;$= −0.253 (65%) $\theta_{Age^{2\;}}$= 0.0579 (145%)
				$\theta_{AGE\prime }=1+\theta_{Age^{1}}\times \left(age-34\right)$	
				IF age >34	
				$\theta_{AGE\prime }=1+\theta_{Age^{2}}\times \left(age-34\right)$	
	+Predictor *Sex at birth*	Kaplan–Meier analysis	Female	$\theta_{SEX\prime }=1+\theta_{Sex\left(\;\right)}$	$\theta_{Sex\left(Male\right)}=0$
					$\theta_{Sex\left(Female\right)}=$0.742 (62%)
	+Predictor *Number of Children*	Kaplan–Meier analysis	Lower number of children	$\theta_{CHILDREN\prime }=1+\theta_{Children}\times \left(children-3\right)$	$\theta_{Children}=$−0.0828 (82%)
	+Predictor Partner’s Viral Load *	*p* < 0.001	Partner’s viral load detectable	-	-
BTS	Baseline (Exponential)	-	-	$\theta_{\lambda }$	0.0005 (23%)
	+Predictor *Sex at birth*	*p* < 0.01	Female	$\theta_{SEX\prime }=1+\theta_{Sex\left(\;\right)}$	$\theta_{Sex\left(Male\right)}=0$
					$\theta_{Sex\left(Female\right)}=$ 2.33 (52%)
	+Predictor *Drug Injection Frequency*	-	Daily drug injection	$\theta_{DRUG\;FREQUENCY\prime }=1+\theta_{Drug\;Frequency\;()}$	$\theta_{Drug\;Frequency\;\left(\;Weekly\right)}$= 0 $\theta_{Drug\;Frequency\;\left(\;Daily\right)}$= 2.22 (67%)
TDF2	Baseline (Exponential)	-	-	$\theta_{\lambda }$	0.0006 (20%)
	No available predictor was found significant.				

* Partner’s viral load covariate was not included in the final model due to missing information in more than 90% of the individuals of partner’s study. $\theta_{PREDICTOR}$ represents the predictor’s model relationship, included in the model as covariate of the baseline hazard parameter (λ). RSE: relative standard error, e.g., for iPrEx study: $\lambda =\theta_{\lambda \;}\times \theta_{RECEPTIVE\prime \;}\times \theta_{AGE\prime \;}\times \theta_{SYPHILIS\prime }$. $\theta_{Predictor\left(\right)}$ represents the parameter value for the categorical predictors, where ( ) can have one category or other. E.g., for sex at birth: $\theta_{Sex\left(\;\right)}$ will take the value of $\theta_{Sex\left(Male\right)}$ or $\theta_{Sex\left(Female\right)}$ depending on the participant’s sex at birth. ↑ Risk: Increase risk.

**Table 3:** Distribution of identified risk factors among low, medium, and high-risk groups.

Study	Participant Risk Factors	Number of Participants with Those Risk Factor	Proportion of Participants with Those Risk Factors in Each Risk Strata		
			Low	Moderate	High
iPrEx	No use of condom when receptive partner	734	7/734 (1%)	257/734 (35%)	470/734 (64%)
	Age < 24	570	0/570 (0%)	238/570 (42%)	332/570 (58%)
	Syphilis positive	178	9/178 (5%)	59/178 (33%)	110/178 (62%)
VOICE	Herpes2 positive	918	197/918 (21%)	316/918 (34%)	405/918 (45%)
	Not being married	1582	67/1582 (4%)	957/1582 (61%)	558/1582 (35%)
	Age < 24	894	50/894 (6%)	427/894 (48%)	417/894 (47%)
	Primary sex partner has sex with another partners	231	55/231 (24%)	58/231 (25%)	118/231 (51%)
	No financial support	348	22/348 (6%)	112/348 (32%)	214/348 (62%)
	No children	304	8/304 (3%)	136/304 (45%)	160/304 (53%)
Partners PrEP	Female	621	61/621 (10%)	501/621 (80%)	59/621 (10%)
	Age < 34	755	51/755 (7%)	645/755 (85%)	59/755 (8%)
	Less than 3 children	619	84/619 (14%)	478/619 (77%)	57/619 (9%)
Bangkok	Female	21	0/21 (0%)	0/21 (0%)	21/21 (100%)
	Daily drug injection	17	0/21 (0%)	0/17 (0%)	21/17 (100%)

## Data Availability

De-identified individual participant data that underlie the results reported in this Article (text, tables, figures, and appendices) will be shared upon request. Proposals and data requests should be directed to Radojka M. Savic (rada.savic@ucsf.edu). Those requesting de-identified data may be required to sign a data access agreement.

## Supplementary Materials

The following supporting information can be downloaded at: https://www.mdpi.com/article/10.3390/pharmaceutics14091801/s1, References [35,36,37,38] are cited in the supplementary materials. Supplementary Material. Additional Methods. Model building and evaluation; Supplementary Table S1 (a–e). Population characteristics and risk factors by studies; Supplementary Material. Preliminary analysis. Figure S1. Kaplan–Meier plots for HIV-free % for iPrEx’ population characteristics; Table S2. Univariate cox-regression analyses for iPrEx study; Figure S2. Kaplan–Meier plots for HIV-free % for VOICE’ population characteristics; Table S3. Univariate cox-regression analyses for VOICE study; Figure S3. Kaplan–Meier plots for HIV-free % for Partners PrEP’ population characteristics; Table S4. Univariate cox-regression analyses for Partners PrEP study; Figure S4. Kaplan–Meier plots for HIV-free % for BTS’ population characteristics; Table S5. Univariate cox-regression analyses for BTS study; Figure S5. Kaplan–Meier plots for HIV-free % for TDF2 population characteristics; Table S6. Univariate Cox-regression analyses for TDF2 study); Table S7. Distribution of identified risk factors (low risk characteristics) among low, medium, and high-risk; Supplementary Material, Additional Results; Supplementary Figure S6. Interactive web application; Supplementary Material, Additional Discussion; Supplementary Material. NONMEM control stream files for the studies.
