# Supplementary material for: Modeling the Probability of HIV Infection over Time in High-Risk Seronegative Participants Receiving Placebo in Five Randomized Double-Blind Placebo-Controlled HIV Pre-Exposure Prophylaxis Trials: A Patient-Level Pooled Analysis
Source: Pharmaceutics. 2022 Aug 27;14(9):1801. doi: 10.3390/pharmaceutics14091801 (PMC9504389; doi:10.3390/pharmaceutics14091801)
Supplement: Supplementary file 1 [file pharmaceutics-14-01801-s001.zip › pharmaceutics-1764582-supplementary.pdf]

SUPPLEMENTAL MATERIAL

Modeling the probability of HIV infection over time in high-risk seronegative individuals receiving placebo in five HIV PrEP trials

Maria Garcia-Cremades, Craig W. Hendrix, Priya Jayachandran Natasha Strydom, Leah Jarlsberg, Robert Grant, Connie L. Celum, Michael Martin, Jared M. Baeten, Jeanne Marrazzo, Peter Anderson, Kachit Choopanya, Suphak Vanichseni, David V. Glidden, Radojka M. Savic

TABLE OF CONTENTS

Supplemental Methods. Model building and evaluation 3

Supplemental Table S1. Population characteristics and risk factors by studies 5

Table S1.(a) Population characteristics and risk factors in iPrEx study 5

Table S1.(b) Population characteristics and risk factors in VOICE study 8

Table S1.(c) Population characteristics and risk factors in Partners PrEP study 11

Table S1.(d) Population characteristics and risk factors in BTS study 13

Table S1.(e) Population characteristics and risk factors in TDF2 study 15

Supplemental Material. Preliminary analysis 16

Figure S1. Kaplan Meier plots for HIV-free % for iPrEx' population characteristics 16

Table S2. Univariate cox-regression analyses for iPrEx study 17

Figure S2. Kaplan Meier plots for HIV-free % for VOICE' population characteristics. 19

Table S3. Univariate cox-regression analyses for VOICE study. 20

Figure S3. Kaplan Meier plots for HIV-free % for Partners PrEP' population characteristics 22

Table S4. Univariate cox-regression analyses for Partners PrEP study. 23

Figure S4. Kaplan Meier plots for HIV-free % for BTS' population characteristics. 24

Table S5. Univariate cox-regression analyses for BTS study. 25

Figure S5. Kaplan Meier plots for HIV-free % for TDF2' population characteristics. 27

Table S6. Univariate cox-regression analyses for TDF2 study. 28

Supplementary Table S7. Distribution of identified risk factors (low risk characteristics) among low, medium, and high-risk……………………………………………………………………………………………26

Supplemental Material. Additional Results. Substantial differences in risk within each population. Detailed description of model predictors in each study. 30

Supplemental Figure S6. Interactive web application 32

Supplemental Material. Additional Discussion. Comparison of the parametric models developed in this work with prior risk assessments tools. 30

Supplemental Material. NONMEM control stream files for the studies……………………………..32

Supplemental Methods.

Model building and evaluation

A parametric time-to-event approach was used to describe time to HIV infection. Different distributions (exponential, Gompertz and Weibull) were explored to characterize the hazard distribution and to select the best baseline survival model. Selection was based on the (i) minimum value of the objective function value (OFV) provided by NONMEM, which is approximately equal to −2 log (likelihood) (−2LL) where a decrease in -2LL of 3.84, 6.63 and 10.83 points between two nested models is considered significant at p values of 0.05, 0.01 and 0.001, respectively, (ii) visual exploratory analysis of predictive checks [20,21] (VPC) and (iii) meaningfulness of the parameter estimates with precision (relative standard errors (RSE)). Once the baseline survival model was selected and evaluated, a covariate analysis was performed to identify predictors ($p < 0.05$) that increased the risk of HIV infection. These predictors could be individual demographic characteristics, risk behaviors, or sexual transmitted infection (STI) history. The covariate identification analysis was performed by stepwise covariate modelling (SCM) implemented in the PsN software [35] by means of the −2LL ratio test with significance level of 0.01 for the forward inclusion and 0.001 for backward deletion approaches. Raw data representation by Kaplan Meier plots were also done in this step to identify potentially significant predictors. Covariates included in the final model were selected with consideration for scientific plausibility, statistical significance and clinical relevance. Selected covariates were included following the general covariate model described in the following equation $TVP = \theta_n \times \prod_1^m \left(\frac{cov_m}{cov_{m,ref}}\right)^{\theta_m} \times \prod_1^p \theta_{p,ctg}$. TVP represents the typical value of a model parameter and is described as a function of m continuous (cov_m) and p categorical covariates. For each p covariate, $\theta_{p,ctg}$ takes the value of 1 for one of the categories (ctg), usually the most common within the studied population, and for the rest of the categories, the corresponding parameter estimates. θ_n describes the nth typical parameter value for an individual with covariate values (cov_m) equal to the reference values: [($cov_m = cov_{m,ref}$) and ($\theta_{p,ctg} = 1$)]. $cov_{m,ref}$ refers to the median value across the populations studied. θ_m is the parameter quantifying the magnitude of the continuous covariate-parameter relationship. Median covariate values

were imputed for missing continuous covariates while categorical missing covariates adopted the most prevalent value of their corresponding population. Potential correlations between covariates were explored through scatter plots and a correlation coefficient calculation. If two highly correlated covariates (>80%) were found, the most significant covariate, in terms of statistical and clinical importance, was chosen and incorporated into the model. If two moderately correlated covariates (>30% and <80%) were found, both were kept in the model in case they showed equivalent significance, provided the evaluation of the final model supported it.

Supplemental Table S1. Population characteristics and risk factors by studies

Table S1.(a) Population characteristics and risk factors in iPrEx study

**iPrEx**	
**Population** – No. (total)	
MSM/TGW	1218
**Demographics**	
Sex at birth	
Male – No. (%)	1218 (100%)
Female – No. (%)	0 (0%)
Ethnicity	
Mixed other – No. (%)	857 (70%)
Black/African American – No. (%)	93 (8%)
White – No. (%)	201 (17%)
Asian – No. (%)	67 (5%)
Country	
Brazil– No. (%)	178 (15%)
Peru– No. (%)	688 (56%)
Ecuador – No. (%)	144 (12%)
USA– No. (%)	113 (9%)
South Africa – No. (%)	39 (3%)
Thailand – No. (%)	56 (5%)
Study site (Location)	
Praca Onze (Brazil) – No. (%)	44 (4%)
USP, Sao Paulo (Brazil) – No. (%)	36 (3%)
Fiocruz (Brazil) – No. (%)	98 (8%)
INMENSA, Lima (Peru) – No. (%)	244 (20%)
ACSA, Iquitos (Peru) – No. (%)	227 (19%)
Equidad (Ecuador)– No. (%)	144 (12%)
SFDPH, San Francisco (USA) – No. (%)	70 (6%)
Fenway Community Health, Boston (USA) – No.	43 (3%)
(%)	39 (3%)
Cape Town (South Africa) – No. (%)	56 (4%)
Chiang Mai (Thailand) – No. (%)	217 (18%)
IMPACTA, Lima (Peru) – No. (%)	
Age (yr)	
Median (min-max)	24 (18-64)
Education	
Less than Secondary – No. (%)	236 (19%)
Completed Secondary – No. (%)	443 (36%)
Post-Secondary – No. (%)	529 (43%)
NA – No. (%)	10 (1%)
Transsexuality	
Yes – No. (%)	158 (13%)
No – No. (%)	1060 (87%)
Marital status	
Single – No. (%)	917 (75%)
With Partner (No.t married) – No. (%)	268 (22%)
Divorced/Separated – No. (%)	15 (1%)
Married – No. (%)	16 (1%)
Widow – No. (%)	2 (<1%)
Weight (Kg)	
Median (min-max)	65.5 (42-177.86)
Latino Ethnicity	
Yes – No. (%)	336 (28%)
No – No. (%)	882 (72%)

Partner report HIV infected	
Yes – No. (%)	31 (3%)
No – No. (%)	1187 (97%)
**<u>Sexual Transmitted Infections</u>**	
HSV2 (herpes simple virus 2)	
Negative – No. (%)	31 (2%)
Positive – No. (%)	421 (35%)
NA – No. (%)	766 (63%)
Urine Luciferase Enzyme Result	
Negative – No. (%)	0 (0%)
Positive 1 – No. (%)	1194 (98%)
Positive 2 – No. (%)	14 (1%)
Positive 3 – No. (%)	6 (<1%)
Positive 4 – No. (%)	3 (<1%)
NA – No. (%)	1 (<1%)
Gonorrhea PCR test	
Negative – No. (%)	18 (1%)
Positive – No. (%)	5 (<1%)
NA – No. (%)	1195 (98%)
Chlamydia PCR test	
Negative – No. (%)	15 (1%)
Positive – No. (%)	8 (<1%)
NA – No. (%)	1195 (98%)
Syphilis RPR test	
Negative – No. (%)	1040 (85%)
Positive – No. (%)	178 (15%)
**<u>Risk behavior</u>**	
Total number of male partners	
Median (min-max)	6 (0-600)
Total number of anal sex partners in the 3 months prior to screening	6 (0-600)
Median (min-max)	
Total number of insertive partners in the past 3 months	
Median (min-max)	2 (0-600)
Total number of receptive partners in the past 3 months	
Median (min-max)	3 (0-600)
Number of non-condomed receptive anal partners	
Median (min-max)	1 (0-300)
Number of non-condomed insertive anal partner	
Median (min-max)	1 (0-102)
Non-condom receptive anal intercourse in the 3 months prior to screening	
Yes – No. (%)	734 (60%)
No – No. (%)	484 (40%)
Total number of condomed receptive anal partners	
Median (min-max)	1 (0-300)
Total number of condomed insertive anal partners	
Median (min-max)	0 (0-600)
Total number of non-condom partners	
Median (min-max)	2 (0-300)
Total number of condom partners	
Median (min-max)	3 (0-600)
Proportion non-condom partners	
Median (min-max)	0.5 (0-2)

No.: Total number of individuals at each group. NA: Information not available.

Table S1.(b) Population characteristics and risk factors in VOICE study

**VOICE**	
**Population** – No. (total)<br>Young women at high risk	2008
Type of placebo	
Oral – No. (%)	1008 (50%)
Vaginal – No. (%)	1000 (50%)
**<u>Demographics</u>**	
Sex at birth	
Male – No. (%)	0 (0%)
Female – No. (%)	2008 (100%)
Ethnicity	
Mixed other – No. (%)	48 (2%)
Black/African American – No. (%)	1960 (98%)
Country	
Zimbabwe– No. (%)	252 (13%)
South Africa – No. (%)	1627 (81%)
Uganda – No. (%)	129 (6%)
Study site (Location)	
Seke South (Zimbabwe) – No. (%)	86 (4%)
Spilhaus (Zimbabwe) – No. (%)	82 (4%)
Zengeza (Zimbabwe) – No. (%)	84 (4%)
Kampala (Uganda) – No. (%)	129 (7%)
Aurum (South Africa) – No. (%)	104 (5%)
Bothas Hill (South Africa) – No. (%)	151 (8%)
eThekwini (South Africa) – No. (%)	144 (7%)
Isipingo (South Africa) – No. (%)	181 (9%)
Overport (South Africa) – No. (%)	98 (5%)
R.K. Khan (South Africa) – No. (%)	167 (8%)
Soweto MTN (South Africa) – No. (%)	140 (7%)
Tongaat (South Africa) – No. (%)	175 (9%)
Umkomaas (South Africa) – No. (%)	146 (7%)
Verulam (South Africa) – No. (%)	180 (9%)
WRHI (South Africa) – No. (%)	141 (7%)
Age (yr)	
Median (min-max)	24 (18-40)
Education	
Less than Secondary – No. (%)	936 (47%)
Completed Secondary – No. (%)	901 (45%)
Post-Secondary – No. (%)	170 (8%)
NA – No. (%)	1 (<1%)
Marital status	
Single – No. (%)	1582 (79%)
Married – No. (%)	426 (21%)
Weight (Kg)	
Median (min-max)	67 (36-157)
Partner circumcised	
Yes – No. (%)	639 (32%)
No – No. (%)	1112 (55%)
Do not know- No. (%)	242 (12%)
NA– No. (%)	15 (1%)
Primary partner report HIV infected	
Yes – No. (%)	63 (3%)
No – No. (%)	1243 (62%)
NA– No. (%)	702 (35%)

Partner provides financial support	
Yes – No. (%)	1646 (82%)
No – No. (%)	348 (17%)
NA – No. (%)	14 (1%)
Number of live birth	
Median (min-max)	1 (0-9)
Alcohol use	
Never – No. (%)	1461 (73%)
<=1x/wk – No. (%)	455 (23%)
2-6x/wk– No. (%)	67 (3%)
Daily – No. (%)	11 (1%)
NA – No. (%)	15 (1%)
**Sexual Transmitted Infections**	
HSV2 (herpes simples virus 2)	
Negative – No. (%)	1082 (54%)
Positive – No. (%)	918 (46%)
NA – No. (%)	8 (<1%)
HSV1 (herpes simples virus 1)	
Negative – No. (%)	86 (4%)
Positive – No. (%)	1914 (96%)
NA – No. (%)	8 (<1%)
Trichomonas test	
Negative – No. (%)	1890 (94%)
Positive – No. (%)	115 (6%)
NA – No. (%)	3 (<1%)
Gonorrhea	
Negative – No. (%)	1939 (97%)
Positive – No. (%)	69 (3%)
Chlamydia	
Negative – No. (%)	1753 (83%)
Positive – No. (%)	255 (13%)
Syphilis	
Negative – No. (%)	1981 (99%)
Positive – No. (%)	27 (1%)
**Risk behavior**	
Vaginal sex past 4 weeks	
Yes – No. (%)	1802 (90%)
No – No. (%)	204 (10%)
NA – No. (%)	2 (<1%)
Used of condom last vaginal sex	
Yes – No. (%)	1470 (73%)
No – No. (%)	278 (14%)
NA – No. (%)	260 (13%)
Total number of vaginal sex in the past 7 days	
Median (min-max)	2 (0-30)
NA – No. (%)	205 (10%)
Type of condom used last vaginal sex	
Male – No. (%)	1237 (62%)
Female – No. (%)	43 (2%)
NA – No. (%)	728 (36%)
Condom used last vaginal sex broke	
Yes – No. (%)	52 (2%)
No – No. (%)	1159 (58%)
NA – No. (%)	797 (40%)

Condom used last vaginal sex put on after penile penetration	
Yes – No. (%)	131 (6%)
No – No. (%)	1036 (52%)
NA – No. (%)	841 (42%)
Condom used last vaginal sex taken off before eyaculation	
Yes – No. (%)	268 (13%)
No – No. (%)	908 (45%)
NA – No. (%)	832 (42%)
Male condom used during last act of anal sex	
Yes – No. (%)	248 (12%)
No – No. (%)	112 (6%)
NA – No. (%)	1648 (82%)
Primary sex partner has sex with another partner in the past 3 months	
Yes – No. (%)	231 (11%)
No – No. (%)	478 (24%)
NA – No. (%)	1299 (65%)
Vaginal sex with primary sex partner in the past 3 months	
Yes – No. (%)	1813 (90%)
No – No. (%)	133 (7%)
NA – No. (%)	62 (3%)
Total number of vaginal sex with condom in the past 7 days	
Median (min-max)	2 (0-30)
NA – No. (%)	592 (30%)
Percentage of condom use in vaginal sex in the past 7 days	
Median (min-max) %	100 (14.29-100)
NA – No. (%)	860 (43%)
Total number of sex partners, excluding primary sex partner, in the past 3 months	
Median (min-max)	0 (0-49)
NA – No. (%)	22 (1%)
Total number of anal sex in the past 3 months	
Median (min-max)	0 (0-7)
NA – No. (%)	29 (1%)

No.: Total number of individuals at each group. NA: Information not available.

Table S1.(c) Population characteristics and risk factors in Partners PrEP study

**Partners PrEP**	
Population – No. (total)	
HIV negative partner in HIV-serodiscordant heterosexual couples	1574
**Demographics**	
Sex at birth	
Male – No. (%)	953 (61%)
Female – No. (%)	621 (39%)
Country	
Kenya– No. (%)	691 (44%)
Uganda– No. (%)	883 (56%)
Study site (Location)	
Tororo (Uganda) – No. (%)	213 (14%)
Nairobi (Kenya)– No. (%)	160 (10%)
Mbale (Uganda) – No. (%)	201 (13%)
Thika (Kenya) – No. (%)	162 (10%)
Kampala (Uganda) – No. (%)	193 (12%)
Kabwohe (Uganda) – No. (%)	175 (11%)
Kisumu (Kenya) – No. (%)	207 (13%)
Eldoret (Kenya) – No. (%)	162 (10%)
Jinja (Uganda) – No. (%)	101 (6%)
Age (yr)	
Median (min-max)	34 (17-64)
Education (years of education at enrollment)	
Median (min-max)	7 (0-19)
Marital status	
Not married – No. (%)	21 (1%)
Married – No. (%)	1553 (99%)
Weight (Kg)	
Median (min-max)	60 (39-164)
Male circumcised (men only)	
Yes – No. (%)	504 (53%)
No – No. (%)	449 (47%)
Currently pregnant (women only)	
Yes – No. (%)	8 (1%)
No – No. (%)	613 (99%)
Any monthly income	
Yes – No. (%)	1250 (79%)
No – No. (%)	324 (21%)
Number of children	
Median (min-max)	3 (0-21)
Viral load (from HIV infected partner)	
Median (min-max)	0 (0-20000000)
NA – No. (%)	1558
Viral load detectable (from HIV infected partner)	
Yes – No. (%)	6 (<1%)
No – No. (%)	6 (<1%)
NA – No. (%)	1562 (99%)
Viral load quantifiable (from HIV infected partner)	
Yes – No. (%)	6 (<1%)
No – No. (%)	10 (1%)
NA – No. (%)	1558 (99%)
**Sexual Transmitted Infections**	

HSV2 (herpes simple virus 2)	
Negative – No. (%)	478 (30%)
Positive – No. (%)	1005 (64%)
NA – No. (%)	91 (6%)
Trichomonas test	
Negative – No. (%)	1453 (92%)
Positive – No. (%)	85 (6%)
NA – No. (%)	36 (2%)
Gonorrhea	
Negative – No. (%)	1488 (95%)
Positive – No. (%)	21 (1%)
NA – No. (%)	65 (4%)
Chlamydia	
Negative – No. (%)	1483 (94%)
Positive – No. (%)	25 (2%)
NA – No. (%)	66 (4%)
Syphilis	
Negative – No. (%)	1500 (95%)
Positive – No. (%)	62 (4%)
NA – No. (%)	12 (1%)
**Risk behavior**	
Number of times had sex with study partner in the last month	
Median (min-max)	4 (0-42)
Number of times used condoms with study partner in the last month	
Median (min-max)	3 (0-42)
NA – No. (%)	59 (4%)
Number of sexual partners besides study partner in last month	
Median (min-max)	0 (0-4)
Number of times had sex with other partners in the last month	
Median (min-max)	4 (1-20)
NA – No. (%)	1454 (92%)
Number of times used condoms with other partners in the last month	
Median (min-max)	0 (0-20)
NA – No. (%)	1454 (92%)

No.: Total number of individuals at each group. NA: Information not available.

Table S1.(d) Population characteristics and risk factors in BTS study

BTS	
Population – No. (total)	
People who inject drugs	177
**Demographics**	
Sex at birh	
Male – No. (%)	156 (88%)
Female – No. (%)	21 (12%)
Country	
Thailand– No. (%)	177 (100%)
Study site (Location)	
Si Phraya, Bangkok (Thailand) – No. (%)	39 (22%)
Urbchit, Bangkok (Thailand)– No. (%)	1 (<1%)
Bang Sue, Bangkok (Thailand) – No. (%)	2 (1%)
Din Daeng, Bangkok (Thailand) – No. (%)	2 (1%)
Wat Thadthong, Bangkok (Thailand) – No. (%)	1 (<1%)
Boonmi, Bangkok (Thailand) – No. (%)	5 (3%)
Wat Pak Boae, Bangkok (Thailand) – No. (%)	1 (<1%)
Prachathipatai, Bangkok (Thailand) – No. (%)	40 (23%)
Ladphrao, Bangkok (Thailand) – No. (%)	38 (21%)
Wongsawang, Bangkok (Thailand) – No. (%)	1 (<1%)
Bangkae, Bangkok (Thailand) – No. (%)	2 (1%)
Khlong Toey, Bangkok (Thailand) – No. (%)	5 (3%)
Wat Paiton, Bangkok (Thailand) – No. (%)	1 (1%)
Vajira, Bangkok (Thailand) – No. (%)	39 (22%)
Age (yr)	
Median (min-max)	30 (35-54)
NA – No. (%)	12 (7%)
Education (years of education at enrollment)	
Less than Secondary – No. (%)	137 (77%)
Completed Secondary – No. (%)	24 (14%)
Post-Secondary – No. (%)	16 (9%)
Weight (Kg)	
Median (min-max)	57 (36.40-93)
NA – No. (%)	15 (8%)
Currently living with partner	
Yes – No. (%)	77 (44%)
No – No. (%)	91 (51%)
NA – No. (%)	9 (5%)
**Risk behavior**	
Use any drugs in last 3 months	
Yes – No. (%)	112 (63%)
No – No. (%)	56 (32%)
NA – No. (%)	9 (5%)
How often injected in last 3 months	
Daily – No. (%)	17 (9%)
Weekly or less often– No. (%)	95 (54%)
NA – No. (%)	65 (37%)
Number of heterosexual sex partners in last 3 months	
Median (min-max)	1 (1-9)
NA – No. (%)	9 (5%)
Frequency of sex with partner	
Never– No. (%)	18 (10%)
Almost never – No. (%)	5 (3%)
50:50 – No. (%)	13 (7%)
Almost always – No. (%)	32 (18%)

Always – No. (%)	9 (5%)
NA – No. (%)	100 (57%)
Condom used with partner	
Never– No. (%)	27 (15%)
Almost never – No. (%)	16 (6%)
50:50 – No. (%)	3 (2%)
Almost always – No. (%)	8 (4%)
Always – No. (%)	5 (3%)
NA – No. (%)	118 (67%)
Condom used with casual partner	
Never– No. (%)	6 (3%)
Almost never – No. (%)	12 (7%)
50:50 – No. (%)	15 (8%)
Almost always – No. (%)	12 (7%)
Always – No. (%)	28 (16%)
NA – No. (%)	104 (59%)
Anal sex in last 3 months	
Yes– No. (%)	13 (7%)
No – No. (%)	134 (75%)
NA – No. (%)	30 (17%)
Number of men had anal sex with	
0-3– No. (%)	7 (4%)
>3 – No. (%)	6 (3%)
NA – No. (%)	164 (93%)
Frequency anal sex with men	
Never– No. (%)	0 (0%)
Almost never – No. (%)	7 (4%)
50:50 – No. (%)	4 (2%)
Almost always – No. (%)	2 (1%)
Always – No. (%)	0 (0%)
NA – No. (%)	164 (93%)
Frequency receptive anal sex	
Never– No. (%)	7 (4%)
Almost never – No. (%)	3 (2%)
50:50 – No. (%)	2 (1%)
Almost always – No. (%)	1 (<1%)
Always – No. (%)	0 (0%)
NA – No. (%)	164 (93%)
Condom used anal sex	
Never– No. (%)	1 (<1%)
Almost never – No. (%)	1 (<1%)
50:50 – No. (%)	4 (2%)
Almost always – No. (%)	1 (<1%)
Always – No. (%)	6 (3%)
NA – No. (%)	164 (93%)
Sex with casual partner last 3 months	
Never– No. (%)	90 (51%)
Almost never – No. (%)	44 (25%)
50:50 – No. (%)	22 (12%)
Almost always – No. (%)	9 (5%)
Always – No. (%)	3 (2%)
NA – No. (%)	9 (5%)

No.: Total number of individuals at each group. NA: Information not available.

Table S1.(e) Population characteristics and risk factors in TDF2 study

**TDF2**	
**Population** – No. (total)	
Heterosexual men & women at high risk	606
**Demographics**	
Sex at birth	
Male – No. (%)	331 (55%)
Female – No. (%)	275 (45%)
Ethnicity	
Black/African American – No. (%)	606 (100%)
Country	
Botswana– No. (%)	606 (100%)
Study site (Location)	
Gaborone (Botswana) – No. (%)	325 (54%)
Francistown (Botswana) – No. (%)	281 (46%)
Age (yr)	
Median (min-max)	24 (18-39)
Education	
Less than Secondary – No. (%)	231 (38%)
Completed Secondary – No. (%)	232 (38%)
Post-Secondary – No. (%)	143 (24%)
Marital status	
Single – No. (%)	568 (94%)
Married – No. (%)	38 (6%)
Weight (Kg)	
Median (min-max)	60 (37.30-136.10)
NA – No. (%)	15 (2%)
**Sexual Transmitted Infections**	
Trichomonas test	
Negative – No. (%)	233 (39%)
Positive – No. (%)	14 (2%)
NA – No. (%)	359 (59%)
Gonorrhea	
Negative – No. (%)	552 (91%)
Positive – No. (%)	12 (2%)
NA – No. (%)	42 (7%)
Chlamydia	
Negative – No. (%)	511 (84%)
Positive – No. (%)	54 (9%)
NA – No. (%)	41 (7%)
Syphilis	
Negative – No. (%)	586 (97%)
Positive – No. (%)	9 (1%)
NA – No. (%)	11 (2%)
**Risk behavior**	
Number of sex partners in the past month	
Median (min-max)	1 (0-10)
NA – No. (%)	11 (2%)
Number of vaginal sex acts (main partner)	
Median (min-max)	5 (0-168)
NA – No. (%)	183 (30%)
Number of vaginal sex acts using condom (main partner)	
Median (min-max)	4 (0-75)
NA – No. (%)	184 (30%)

No.: Total number of individuals at each group.

Supplemental Material. Preliminary analysis

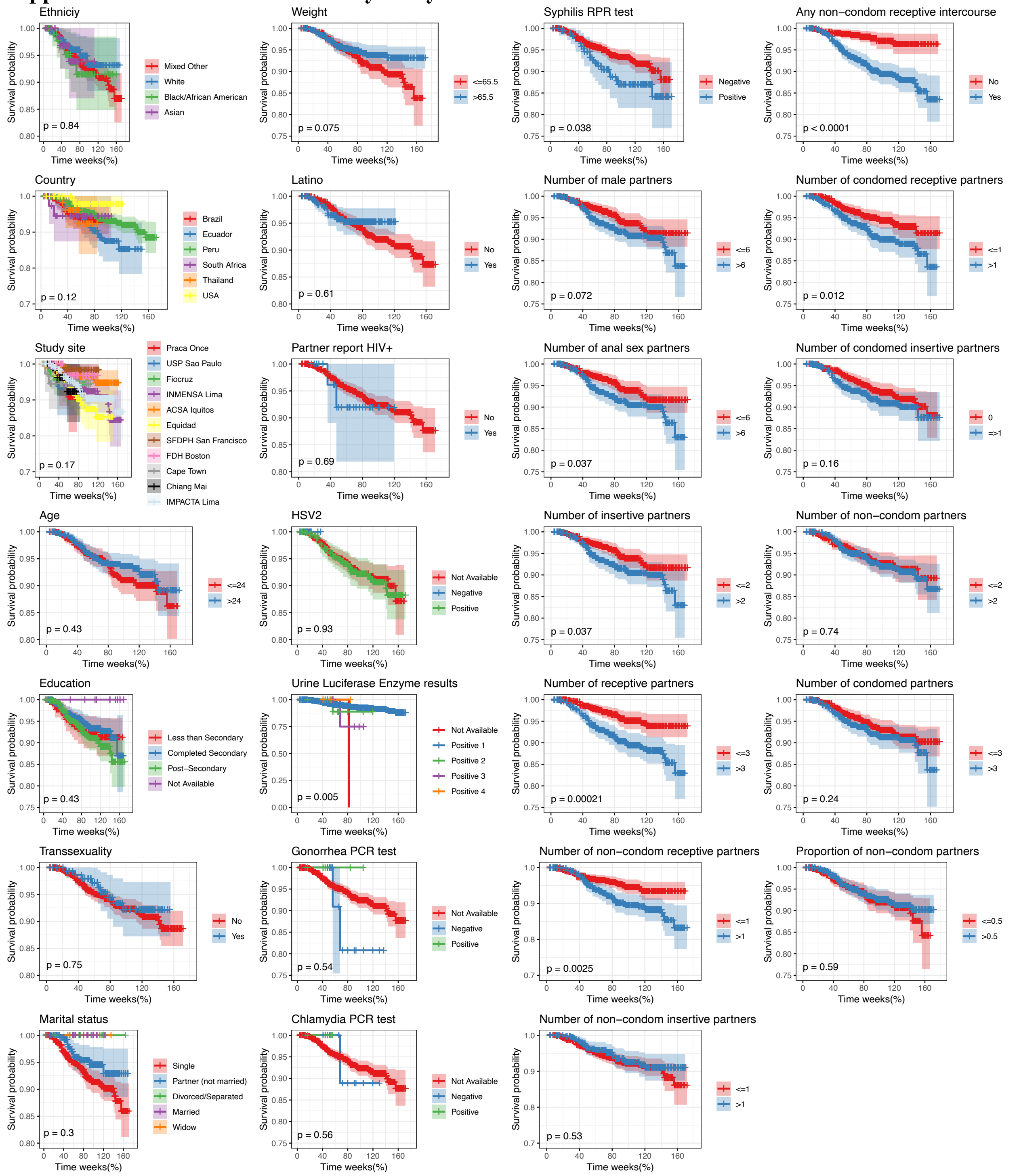

Figure S1. Kaplan Meier plots for HIV-free % for iPrEx' population characteristics. *Sexual risk behavior characteristics are accounted from the past 3 moths to baseline.

Table S2. Univariate cox-regression analyses for iPrEx study.

	beta	HR (95% CI for HR)	p.value
Ethnicity (reference Mixed/Other)			
White	-0.29	1.33 (0.37-1.51)	0.423
Black/African American	0.15	1.16 (0.5-2.68)	0.735
Asian	0.05	1.05 (0.33-3.39)	0.931
Age (years)	-0.024	0.98 (0.95-1)	0.11
Education (reference Less than Secondary)			
Completed Secondary	-0.18	0.84 (0.45-1.56)	0.579
Post-Secondary	0.15	1.17 (0.65-2.08)	0.6
Not available	-15	2.9E-07 (0-Inf)	0.994
Transsexuality (reference No)			
Yes	-0.11	0.9 (0.46-1.74)	0.753
Marital status (reference Single)			
Partner (not married)	-0.5	0.61 (0.33)	0.11
Divorced/Separated	-16.15	9.7E-08 (0-Inf)	0.996
Married	-16.15	9.7E-08 (0-Inf)	0.996
Widow	-16.15	9.7E-08 (0-Inf)	0.998
Weight (Kg)	-0.014	0.99 (0.97-1)	0.131
Latino (reference No)			
Yes	-0.15	0.86 (0.48- 1.55)	0.609
Partner report HIV + (reference No)			
Yes	0.29	1.3 (0.33-5.4)	0.69
HSV2 (reference Negative)			
Positive	14.04	1.2E+6 (0-Inf)	0.997
Urine Luciferase Enzyme result (reference Positive 1)			
Positive 2	0.43	1.54 (0.21-11.09)	0.669
Positive 3	1.01	2.74 (0.38-19.73)	0.317
Positive 4	-13.01	2.24E-06 (0-Inf)	0.994
Gonorrhea PCR test (reference Negative)			
Positive	-19.3	4.23E-09 (0-Inf)	0.999
Chlamydia PCR test (reference Negative)			
Positive	1.35	3.87 (0.24-62.4)	0.34
Syphilis RPR (reference Negative)			
Positive	0.53	1.7 (1-2.8)	0.04
Number of male partners	0.0016	1 (1-1)	0.479
Number of anal sex partners	0.002	1 (1-1)	0.4
Number of insertive partners	-0.0011	1 (0.99-1)	0.85
Number of receptive partners	0.0041	1 (1-1)	0.083
Number of non-condom receptive partners	0.008	1 (1-1)	0.089
Number of non-condom insertive partners	-0.022	0.98 (0.94-1)	0.27
Any non-condom receptive intercourse in the 3 months prior to screening (reference No)			
Yes	1.36	3.9 (2.1-7.4)	2.59E-05
Number of condomed receptive partners	0.0048	1 (1-1)	0.25
Number of condomed insertive partners	0.0012	1 (0.99-1)	0.77
Number of non-condom partners	0.0033	1 (0.99-1)	0.5
Number of condomed partners	0.0022	1 (1-1)	0.37
Proportion of non-condom partners	-0.11	0.89 (0.55-1.5)	0.65

p.value reported for Wald test in univariate cox regression analysis

Missing values were excluded from this analysis

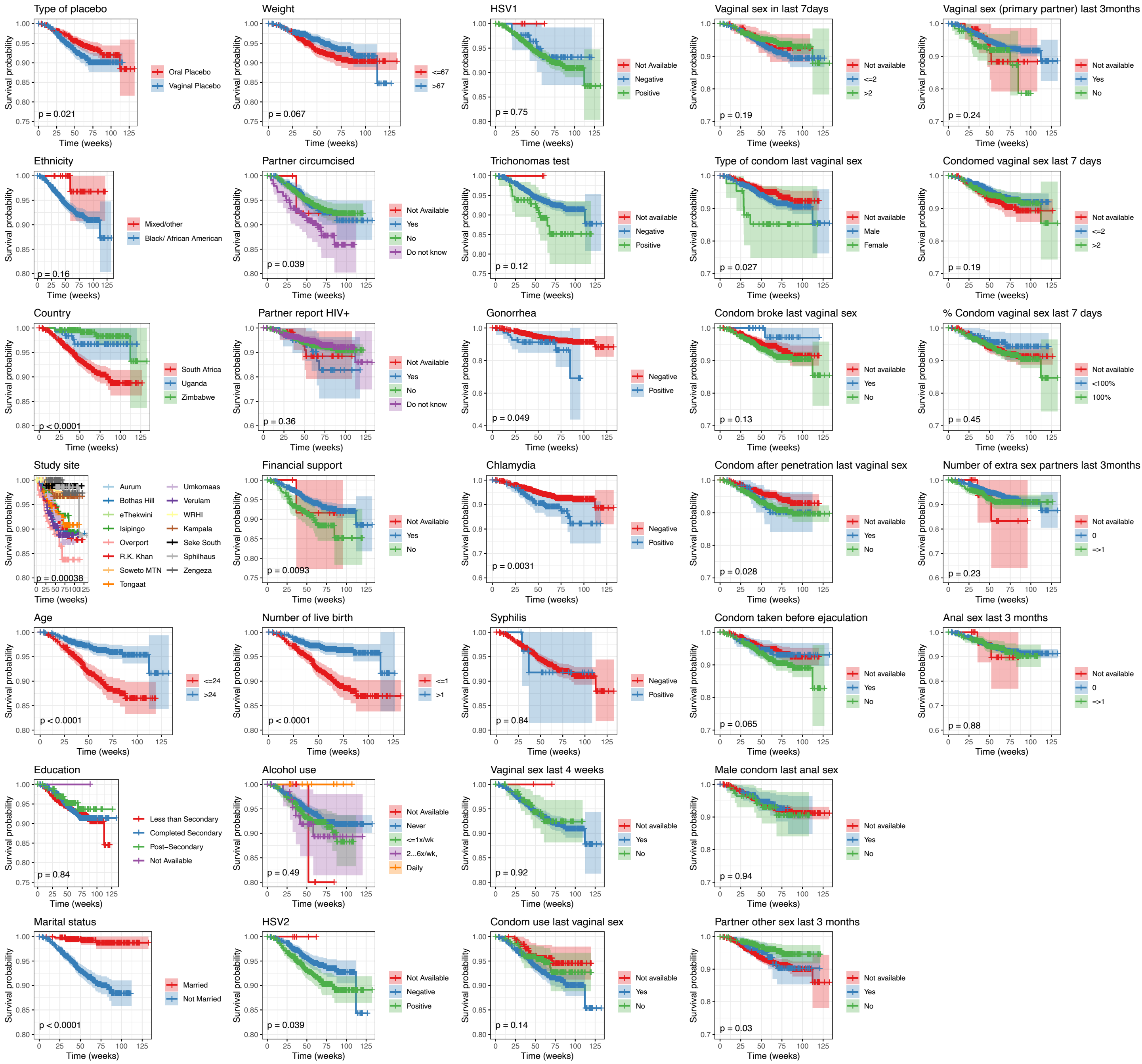

Figure S2. Kaplan Meier plots for HIV-free % for VOICE' population characteristics.

Table S3. Univariate cox-regression analyses for VOICE study.

	beta	HR (95% CI for HR)	p.value
Type of placebo (reference Oral)			
Vaginal	0.41	1.5 (1.1-2.13)	0.02
Ethnicity (reference Mixed/other)			
Black/ African American	1.3	3.755 (0.52-26.89)	0.2
Country (reference South Africa)			
Uganda	-1.1	0.34 (0.12-0.92)	0.03
Zimbabwe	-1.79	0.17 (0.06-0.46)	4.80E-04
Age (years)	-0.12	0.88 (0.85-0.92)	1.80E-08
Education (reference Less than Secondary)			
Completed Secondary	-0.05	0.95 (0.66-1.36)	0.774
Post-Secondary	-0.32	0.73 (0.35-1.52)	0.397
Marital status (reference Married)			
Not married	2.33	10.29 (3.78-28.02)	5.08E-06
Weight (kg)	-0.014	0.99 (0.98-1)	0.02
Partner circumcised (reference Yes)			
No	-0.4	1.04 (0.64-1.43)	0.84
Do not know	0.59	1.8 (1.1-3)	0.02
Partner report HIV+ (reference Yes)			
No	-0.42	1.68 (0.27-1.29)	0.19
Do not know	-0.66	1.93 (0.23-1.6)	0.11
Financial support (reference Yes)			
No	0.6	1.83 (1.24-2.7)	0.0026
Number of live birth (children)	-0.47	0.62 (0.52-0.75)	1.10E-06
Alcohol use (reference Never)			
Weekly or less	0.27	1.31 (0.89-1.94)	0.173
2-6 times per week	0.41	1.5 (0.66-3.44)	0.332
Daily	-14.9	3.2E-07 (0-Inf)	0.995
HSV2 (reference Negative)			
Positive	0.4375	1.55 (1.09-2.19)	0.014
HSV1(reference Negative)			
Positive	0.25	1.28 (0.52-3.13)	0.59
Trichonomas test (reference Negative)			
Positive	0.58	1.79 (1.01-3.18)	0.046
Gonorrhea (reference Negative)			
Positive	0.7	2.02 (0.99-4.14)	0.054
Chlamydia (reference Negative)			
Positive	0.63	1.88 (1.23-2.87)	0.004
Syphilis (reference Negative)			
Positive	0.15	1.16 (0.29-4.7)	0.835
Vaginal sex last 4 weeks (reference Yes)			
No	-0.06	0.95 (0.52-1.71)	0.854
Condom use last vaginal sex (reference Yes)			
No	-0.31	0.73 (0.43-1.26)	0.256
Number of vaginal sex in last 7 days	-0.08	0.92 (0.85-1)	0.057
Type of condom last vaginal sex (reference Male)			
Female	0.76	2.14 (0.94-4.9)	0.071
Condom used last vaginal sex broke (reference Yes)			

No	1.4	4.04 (0.56-29)	0.165
Condom last vaginal sex put on after penile penetration (reference Yes)			
No	-0.08	0.92 (0.49-1.73)	0.803
Condom used last vaginal sex taken off before ejaculation (reference Yes)			
No	0.35	1.42 (0.81-2.47)	0.22
Male condom used during last anal sex (reference Yes)	0.17	1.19 (0.5-2.8)	0.69
No			
Primary sex partner has sex with another partner last 3 months (reference Yes)			
No	-0.57	0.57 (0.29-1.13)	0.106
Vaginal sex with primary sex partner last 3 months (reference Yes)			
No	0.46	1.58 (0.87-2.86)	0.133
Number of Condomed vaginal sex last 7 days	0.017	1.02 (0.95-1.09)	0.643
%Condom use in vaginal sex last 7 days	0.01	1.01 (0.995-1.03)	0.174
Number of sex partners (excluding primary sex partner) with whom had vaginal sex in the past 3 months	-0.02	0.98 (0.88-1.09)	0.727
Number of anal sex in the past 3 months	-0.04	0.96 (0.83-1.11)	0.572

p.value reported for Wald test in univariate cox regression analysis
Missing values were excluded from this analysis

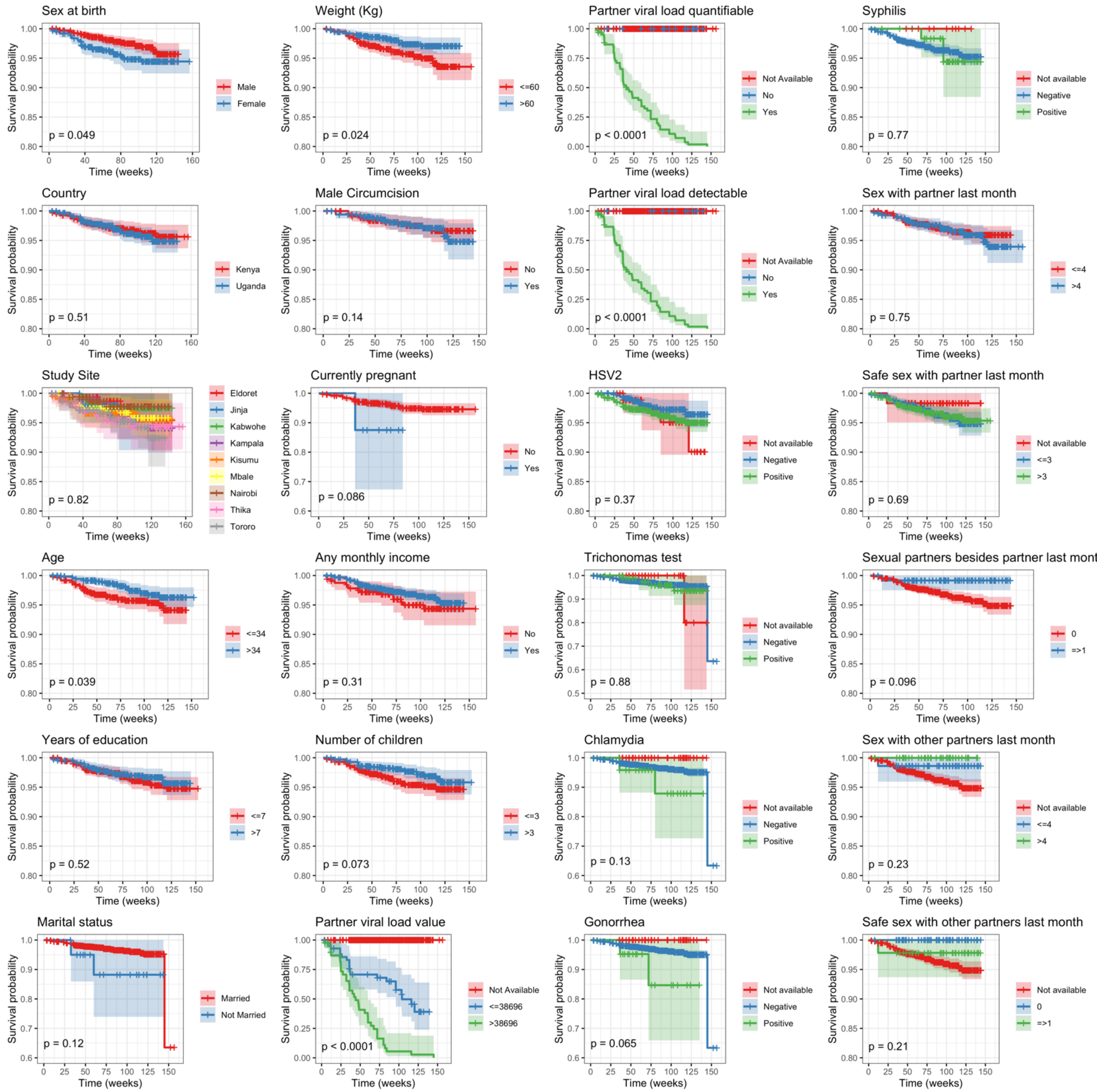

Figure S3. Kaplan Meier plots for HIV-free % for Partners PrEP' population characteristics. Individual characteristics shown are baseline measurements instead of Partner viral load value, Partner viral load quantifiable and Partner viral load detectable, which last values before HIV infection or censor are used in this plot representation.

Table S4. Univariate cox-regression analyses for Partners PrEP study.

	beta	HR (95% CI for HR)	p.value
Sex at birth (reference Male)			
Female	0.52	1.7 (1-2.8)	0.052
Age (years)	-0.057	0.94 (0.91-0.98)	0.0013
Years of education	-0.054	0.95 (0.89-1)	0.103
Marital status (reference Married)			
Not Married	1.07	2.9 (0.71-11.98)	0.137
Weight	-0.035	0.97 (0.94-0.99)	0.022
Male Circumcision* (reference No)			
Yes	0.119	1.13 (0.53-2.41)	0.758
Currently Pregnant** (reference No)			
Yes	1.171	3.23 (0.44-23.81)	0.251
Monthly Income (reference No)			
Yes	-0.302	0.74 (0.41-1.34)	0.317
Number of children	-0.137	0.87 (0.78-0.98)	0.021
Partner viral load value	3.59E-08	1 (1-1)	0.229
Partner viral load quantifiable (reference No)			
Yes	20.17	5.7E+08 (0-Inf)	0.995
Partner viral load detectable (reference No)			
Yes	19.83	4.1E+08 (0-Inf)	0.996
HSV2 (reference Negative)			
Positive	0.434	1.54 (0.81-2.94)	0.188
Trichomona test (reference Negative)			
Positive	0.251	1.29 (0.46-3.56)	0.629
Chlamydia (reference Negative)			
Positive	0.897	2.5 (0.6-10.06)	0.213
Gonorrhea (reference Negative)			
Positive	1.175	3.24 (0.79-13.31)	0.103
Syphilis (reference Negative)			
Positive	0.156	1.17 (0.37-3.75)	0.792
Sex with partner last month (frequency)	0.019	1.02 (0.97-1.07)	0.405
Safe sex with partner last month (frequency)	0.0034	1 (0.99-1.02)	0.656
Sexual partners besides regular partner last month	-1.44	0.24 (0.04-1.62)	0.14
Sex with other partners last month*** (frequency)	-17.9	1.69E-08 (0-Inf)	0.998
Safe sex with other partners last month*** (frequency)	-0.68	0.93 (0.37-2.35)	0.886

p.value reported for Wald test in univariate cox regression analysis
Missing values were excluded from this analysis
**Including only males at birth*
*** Including only females at birth*
**** Including only those participants who have sexual partners besides regular partner*

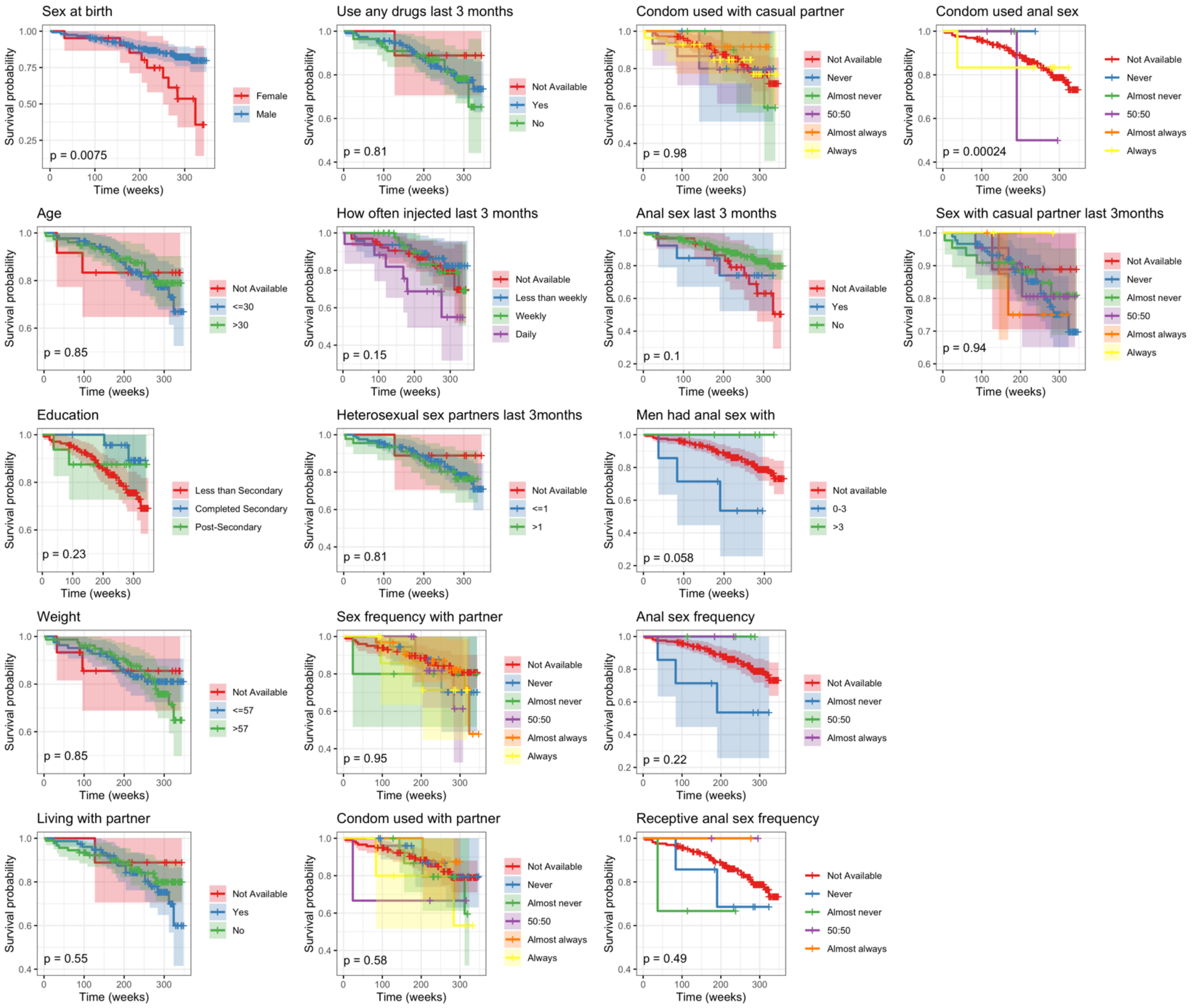

Figure S4. Kaplan Meier plots for HIV-free % for BTS' population characteristics.

Table S5. Univariate cox-regression analyses for BTS study.

	beta	HR (95% CI for HR)	p.value
Sex at birth (reference Female)			
Male	-1	0.37 (0.17-0.79)	0.0103
Age (years)	-0.015	0.99 (0.95-1.03)	0.478
Education (reference Less than secondary)			
Completed Secondary	-1.13	0.32 (0.08-1.36)	0.124
Post-Secondary	-0.46	0.63 (0.15-2.63)	0.525
Weight (Kg)	0.004	0.996 (0.96-1.03)	0.799
Living with partner (reference Yes)			
No	-0.315	0.73 (0.36-1.46)	0.375
Use any drugs last 3 months (reference Yes)			
No	0.072	1.07 (0.52-2.24)	0.847
How often injected last 3 months (reference Less than weekly)	0.3	1.35 (0.49-3.74)	0.559
Weekly	1.2	3.33 (1.11- 9.96)	0.032
Daily			
Heterosexual sex partners last 3months	0.012	1.01 (0.88-1.17)	0.865
Sex frequency with partner (reference Never)			
Almost never	0.007	1.01 (0.11-9.03)	0.995
50:50	0.211	1.23 (0.27-5.66)	0.786
Almost always	-0.074	0.93 (0.27-3.19)	0.906
Always	0.191	1.21 (0.22-6.73)	0.827
Condom used with partner (reference Never)			
Almost never	0.679	1.97 (0.53-7.36)	0.313
50:50	0.977		0.385
Almost always	-0.222		0.843
Always	0.829		0.346
Condom used with casual partner (reference Never)			
Almost never	0.302	1.35 (0.14-13.01)	0.794
50:50	0.178	1.19 (0.12-11.53)	0.878
Almost always	-0.69	1.5 (0.03-8.03)	0.626
Always	0.163	1.18 (0.14-10.15)	0.882
Anal sex last 3 months (reference Yes)			
No	-0.604	0.55 (0.16-1.84)	0.33
Men had anal sex with (Reference 0.3)			
>3	-21.1	6.48E-10 (0-Inf)	0.999
Anal sex frequency (reference Almost never)			
50:50	-21.1	6.48E-10 (0-Inf)	0.999
Almost always	-21.1	6.51E-10 (0-Inf)	1
Receptive anal sex frequency (reference Never)			
Almost never	0.52	1.69 (0.15-19.18)	0.66
50:50	-19.36	3.89E-09 (0-Inf)	0.999
Almost always	-19.37	3.86E-09 (0-Inf)	0.999
Condom used anal sex frequency (reference Never)			
Almost never	-0.024	0.98 (0-Inf)	1
50:50	19.06	1.89E+08 (0-Inf)	0.999
Almost always	21	1.32E+09 (0-Inf)	0.999
Always	18.44	1.02E+08 (0-Inf)	0.999
Sex with casual partner last 3months frequency (reference Never)	-0.247	0.78 (0.33-1.87)	0.58
Almost never	-0.156	0.86 (0.29-2.53)	0.778
50:50	0.289	1.34 (0.31-5.78)	0.698
Almost always	0.465	1.59 (0.21-11.96)	0.651
Always			

p.value reported for Wald test in univariate cox regression analysis

Missing values were excluded from this analysis

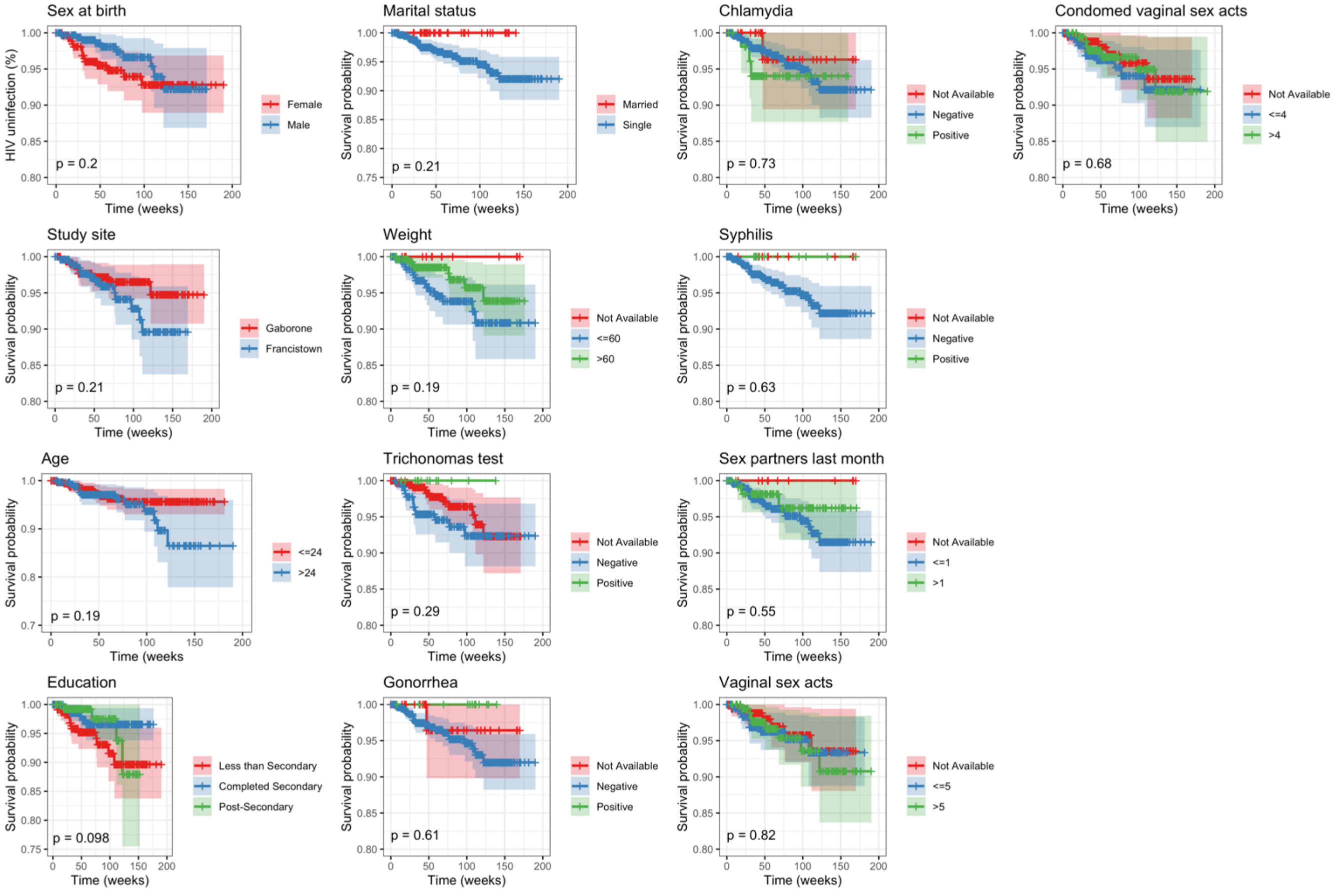

Figure S5. Kaplan Meier plots for HIV-free % for TDF2’ population characteristics.

Table S6. Univariate cox-regression analyses for TDF2 study.

	beta	HR (95% CI for HR)	p.value
Sex at birth (reference Female)			
Male	-0.52	0.59 (0.26-1.3)	0.209
Study site (reference Gaborone)			
Francistown	0.515	1.67 (0.74-3.77)	0.214
Age	0.0297	1.03 (0.92-1.15)	0.591
Education (reference Less than Secondary)	-0.941	0.39 (0.15-1.02)	0.054
Completed Secondary	-0.725	0.48 (0.16-1.47)	0.201
Post-Secondary			
Marital status (reference Married)			
Single	17.1	2.7E+07 (0-Inf)	0.999
Weight (Kg)	-0.039	0.96 (0.92-1)	0.06
Trichomonas test (reference Negative)			
Positive	-17.1	3.8E-08 (0-Inf)	0.998
Gonorrhea (reference Negative)			
Positive	-16.1	1.07E-07 (0-Inf)	0.997
Chamydia (reference Negative)			
Positive	0.379	1.46 (0.43-4.92)	0.54
Syphilis (reference Negative)			
Positive	-16.03	1.1E-07 (0-Inf)	0.997
Sex partners last month	-0.23	0.77 (0.44-1.35)	0.363
Vaginal sex acts	-0.015	0.98 (0.92-1.05)	0.655
Condomed vaginal sex acts	-0.032	0.97 (0.89-1.06)	0.475

p.value reported for Wald test in univariate cox regression analysis
Missing values were excluded from this analysis

Supplementary Table S7. Distribution of identified risk factors (low risk characteristics) among low,

Study	Participant risk factors	Number of participants with those risk factor	Proportion of participants with those risk factors in each risk strata		
			Low	Moderate	High
iPrEx	Use of condom when receptive partner	484	83/484 (17%)	401/484 (83%)	0/484 (0%)
	Age ≥24	648	90/648 (14%)	420/648 (65%)	138/648 (21%)
	Syphilis -	1040	81/1040 (7%)	599/1040 (58%)	360/1040 (34%)
VOICE	Herpes2 -	1082	256/1082 (24%)	679/1082(63%)	147/1082 (13%)
	Being married	426	388/426 (91%)	38/426 (9%)	0/426 (0%)
	Age ≥24	1114	405/1114 (36%)	568/1114 (51%)	141/1114 (13%)
	Primary sex partner does not have sex with another partners	1777	400/1777 (23%)	937/1777 (53%)	440/1777 (25%)
	Receive financial support	1646	432/1646 (26%)	877/1646 (53%)	337/1646 (20%)
	1 child or more	1704	447/1704 (26%)	859/1704 (50%)	398/1704 (23%)
Partners PrEP	Male	953	424/953 (44%)	529/953 (56%)	0/953 (0%)
	Age ≥34	819	434/819 (53%)	385/819 (47%)	0/819 (0%)
	More than 3 children	955	401/955 (42%)	552/955 (58%)	2/955 (0.1%)
Bangkok	Male	156	0/156 (0%)	139/156 (89%)	17/156 (11%)
	Weekly or less frequency drug injection	95	0/95 (0%)	78/95 (82%)	17/95 (18%)

medium, and high-risk.

Supplemental Material. Additional Results.

<u>Substantial differences in risk within each population. Detailed description of model predictors in each study.</u>

For the *MSM/TGW* population, age, syphilis seroreactivity and receptive anal intercourse without a condom in the 3 months before screening were identified as predictors of the baseline hazard during the forward inclusion ($p<0.05$) of the SCM analysis; therefore, these predictors were incorporated in the final multivariate parametric model. The inclusion of syphilis seroreactivity showed a $p>0.05$ and a high RSE of its corresponding parameter (Table II), probably due to a misrepresentation of the syphilis positive individuals in the current dataset (15%) (Table SIa). Nevertheless, syphilis seroreactivity was kept as a predictor in the final model (supported by the Kaplan Meier plot of the raw data (Figure S1)). Receptive anal intercourse without a condom was the strongest predictor of HIV infection among MSM/TGW, with a calculated HR of 5.88, while younger participants (20 years old) had a HR of 1.12 and being syphilis positive had a HR of 1.64.

For the *young women at high sexual risk* int the VOICE trial, herpes simplex virus 2 (HSV2), marital status, age, and financial support provided by a partner were identified as significant predictors by the SCM analysis ($p<0.05$). The number of children was also incorporated in the model, which was supported by the Kaplan Meier plot of the raw data (Figure S2) and the univariate Cox regression analysis (Table S3). The last predictor incorporated in the model was if the primary sex partner has sex with another partner in the past 3 months, also supported by the Kaplan Meier plot of the raw data. Interestingly, those individuals with missing information (65%) showed a similar risk of HIV infection as those whose primary sex partner has sex with another partner (Figure S2). Therefore, the individuals with missing information were included in the same category as 'primary sex partner has sex with another partner'. Not being married was the strongest predictor of HIV infection in young women in the VOICE trial (HR=5.34), followed by HSV2 seropositivity (HR=1.99), if her partner has another sex partner (HR=1.79), if her partner does not provide financial support (HR=1.51), younger individuals (HR=1.2 for 21 years old), and has no children (HR=1.16).

For the *HIV negative partners in HIV-serodiscordant heterosexual couples (Partners PrEP)*, age and partner's viral load were identified as significant predictors by the SCM analysis; however, the latter was not retained in the multivariate model due to missing information in more than 90% of the individuals. Sex and number of children were included in the final multivariate model guided by the Kaplan Meier plots of the raw data (Figure S3). Age was included using a piece-wise linear function, where younger participants were at a higher risk (HR=2.52 for 28 years old), but at a specific age (34 years old, population median), the risk starts increasing with age (HR=1.35 for 40 years old). Women had a HR of 1.74 and women with no children has a HR of 1.25.

For the *people who inject drugs (BTS)*, sex and drug injection frequency were identified and included as significant predictors in the final multivariate model ($p<0.01$). For drug injection frequency, two categories included in the final model were *daily* and *weekly or less than weekly*. There were three categories in the BTS: *daily*, *weekly*, and *less than weekly*; however, there was no significant difference in weekly and less than weekly, and we combined these categories. Higher risk was observed in female participants (HR=3.33) and in those who use drugs daily (HR=3.22).

No predictors ($p<0.05$) were identified in the TDF2 study to be included in the model; therefore, only the baseline hazard risk for the entire population was characterized.

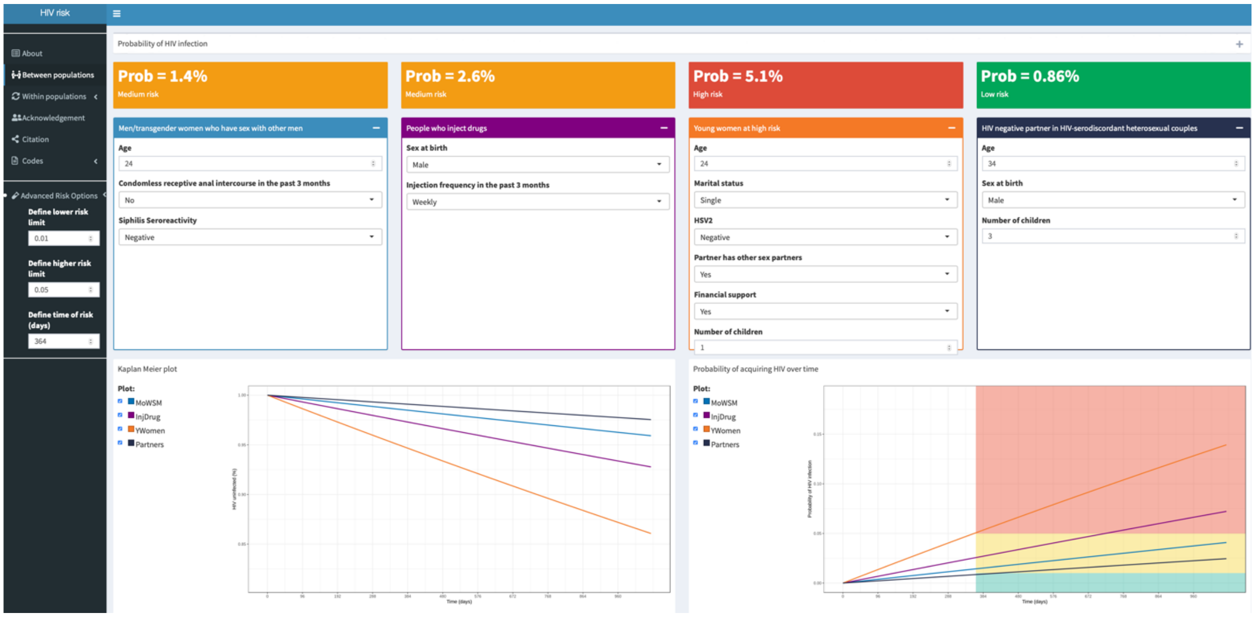

Supplemental Figure S6. Interactive web application. A page of the web app is shown that displays the probability of being HIV uninfected for the different key population based on the individual characteristics and risk factors.

Supplemental Material. Additional Discussion.

Comparison of the parametric models developed in this work with prior risk assessments tools.

The findings from the parametric models contribute to and are in agreement with the findings from prior risk assessment tools developed to predict HIV infection [14,16,17,27-31].

For the *MSM/TGW population*, not using condoms as a receptive partner has been identified as a predictor of HIV infection [17, 31]. Self-reported STI, specifically baseline syphilis seroreactivity, was identified as a predictor of HIV infection in a univariate analysis with a similar HR to HR in our study (1.58 vs 1.64, respectively) [31]. Smith et al. identified age as a significant predictor [7]. While several studies reported drug use as a significant predictor [17, 31], that predictor was unavailable in our database. Among *African women*, age, marital status, financial support provided by a partner, if the partner has another partner, HSV2 status, and alcohol consumption were included in a risk scoring tool [15], analyzing data from 3 RCTs [3,7,36] including VOICE. In contrast to our analysis, this assessment also included information from the treatment arms, which may act as a confounding factor in determining the true effect of each predictor on HIV risk. In the parametric model for this population, alcohol consumption was not significant during model building. Similarly, a risk assessment tool was developed for identifying couples at highest risk of HIV transmission [14] that included data from three studies in Africa of stable *serodiscordant couples* [4, 37,38] and the placebo arm of the Partners PrEP study as a validation cohort. Kahle et al. identified age, marital status, number of children, unprotected sex, uncircumcised male, and a partner's viral load as significant predictors. In contrast, our analysis found no significance for male circumcision (Figure S3) and marital status (99% married). Although information regarding safe sex and partner viral load was available, it was missing for approximately 90% of the individuals; even though these variables were found to be significant in sub cohort analysis, they were not used in the main analysis. As an example, among the 85 of 1574 individuals with viral load information available for a partner, 100% of the participants who had partners with quantifiable viral load (n=49) were acquired HIV during the follow up, and among participants who had partners with undetectable viral loads (n=36) none acquired HIV. This is consistent with studies indicating essentially no sexual HIV transmission risk when the viral load of the sexual partner is below detection limits [33]. For the *people who inject drugs* population, needle sharing was described

in the BTS study [5] as an important risk factor of HIV infection; however, that information was unavailable for analysis.

Supplemental Material. NONMEM control stream files for the studies

<u>NONMEM control stream file for iPrEx study</u>

```
;; 1. Based on: run9
;; 2. Description: OS-EXP. Placebo. STUDY1

$PROBLEM    EXP_MODEL HIV_SURVIVAL PLACEBO

$INPUT ID PTT=DROP PTLAST=DROP PTMED=DROP PBTMED=DROP PTLMED=DROP PTLMED=DROP
TTLAST=DROP TTFVMED=DROP TTLMED=DROP FLAGTDP=DROP TIME PRODUCT     HIVF HIV
CW=DROP TOT=DROP AN=DROP TF=DROP BF=DROP NCP=DROP NCIAP=DROP NCRAI=DROP
PRAI=DROP PIAI=DROP UPP=DROP PP=DROP PRU=DROP HSV=DROP URN=DROP URNG=DROP
URCL=DROP SYP SYP2=DROP PDFLAG SCCRAI SCRPAR=DROP LAT=DROP RACE=DROP AGE
EDU=DROP RINF=DROP SITE=DROP TRANS STUDY SEX MARST HEIGHT WEIGHT CRCL SECR EVID
DV FLAG_SIMUL DATHIV OFFD=DROP TVBI=DROP CTBI=DROP GCBI=DROP COHOR=DROP
TVBL=DROP CTBL=DROP GCBL=DROP SYPH3=DROP SYPPH4=DROP PSEXF=DROP PSAF=DROP
OTHP=DROP OSFR=DROP OSAFR=DROP HSV2BL=DROP HSV2=DROP BLCR=DROP PREG MINC=DROP
HIVA=DROP VIS=DROP GON=DROP CT=DROP HSV11=DROP HSV22=DROP HSV1W=DROP
HSV2W=DROP TV=DROP SECRE2=DROP

$DATA        prepHIV_PD_final_180411.csv
IGNORE (FLAG_SIMUL.EQ.1)
IGNORE (PRODUCT.LT.10)
IGNORE (STUDY.NE.1)

$SUBROUTINE ADVAN13 TOL=9
$MODEL
COMP (HAZ)

;;time-date (weeks!)
;;dv- hiv
;; Data for estimation FLAG_SIMUL=0
;; Data for simulation FLAG=0 + FLAG=1
;; PRODUCT LT 10= PLACEBO
$PK

;;; BASESCCRAI-DEFINITION START
IF(SCCRAI.EQ.1) BASESCCRAI = 1  ; Most common
IF(SCCRAI.EQ.0) BASESCCRAI = ( 1 + THETA(2))
;;; BASESCCRAI-DEFINITION END

;;; BASEAGE-DEFINITION START
BASEAGE = ( 1 + THETA(3)*(AGE - 24))
;;; BASEAGE-DEFINITION END

;;; BASESYP-DEFINITION START
IF(SYP.EQ.0) BASESYP = 1  ; Most common
IF(SYP.EQ.1) BASESYP = ( 1 + THETA(4))
;;; BASESYP-DEFINITION END

;;; BASE-RELATION START
BASECOV=BASESCCRAI*BASEAGE*BASESYP
;;; BASE-RELATION END

;--------DROPOUT MODEL-----------
A_0(1)=0
TVBASE=THETA(1)
```

```
TVBASE = BASECOV*TVBASE
BASE=TVBASE*EXP(ETA(1))

$DES

;--------DROPOUT MODEL-----------
DEL=1E-6
DADT(1)=BASE

$ERROR
;--------DROPOUT MODEL-----------
CHZ = A(1)        ;cumulative hazard
SUR = EXP(-CHZ)          ;survival probability
DEL2=1E-6
HAZNOW=BASE

IF (DV.EQ.0) Y=SUR
IF (DV.EQ.1) Y=SUR*HAZNOW

$THETA
(0, 0.00113)              ; BASE
(-1, -0.743,5)            ; BASESCCRAI1
(-0.1, -0.0273,0.167) ; BASEAGE1
(-1,-0.1,5)               ; BASESYP1

$OMEGA 0 FIX      ;     BASE

$ESTIMATION METHOD=0 LIKE MAXEVAL=9900 NOABORT PRINT=1 SIGL=9 NSIG=3 MSFO=msfb522
$COV PRINT=E
$TABLE ID TIME DV SUR CHZ FORMAT=s1PE15.8 ONEHEADER NOPRINT FILE=sdtab22
```

NONMEM control stream file for VOICE study

```
;; 1. Based on: run30_voicf
;; 2. Description: with placebo_voice3_risksex_ST3

$PROBLEM    EXP_MODEL HIV_SURVIVAL PLACEBO

$INPUT ID PLASMA_TFV_LAST=DROP PBMC_TFVDP_LAST=DROP TISSUE_TFV_LAST=DROP
PLASMA_TFV_MED=DROP PBMC_TFVDP_MED=DROP TISSUE_TFV_ME=DROP
PLASMA_TFV_LAST_MED=DROP PBMC_TFVDP_LAST_MED=DROP TISSUE_TFV_LAST_MED=DROP
FLAG_TFV_TFVDP=DROP TIME PRODUCT hiv_final=DROP hiv=DROP hiv_acute=DROP PD_flag=DROP
visit=DROP syph gon ct hsv1 HSV22 hsv1wb hsv2wb tv height secre=DROP crcl=DROP wt_kg sex MARST
education AGE CIRC CHILD FIN race=DROP C_3mvs C_4wvs C_7dvsn C_7dvscn C_lvsc C_lvsct C_7dpvsc
A_7dvsn A_7dvscn A_lvsc A_lvsct A_lvscb A_lvsca A_lvsce A_3masn A_lasmc A_3MPPSO A_3mvspp
A_3mvsnp Q5 Q12 study=DROP EVID DV FLAG_SIMUL

$DATA    Voice_PD_180919.csv
IGNORE (FLAG_SIMUL.EQ.1)
IGNORE (PRODUCT.LT.10)

$SUBROUTINE ADVAN13 TOL=9

$MODEL
COMP (HAZ)

;;time-date (weeks!)
;;dv- hiv
;; Data for estimation FLAG_SIMUL=0
;; Data for simulation FLAG=0 + FLAG=1
;; PRODUCT LT 10= PLACEBO

$PK

;;; BASEHSV22-DEFINITION START
IF(HSV22.EQ.0) BASEHSV22 = 1  ; Most common
IF(HSV22.EQ.1) BASEHSV22 = ( 1 + THETA(2))
IF(HSV22.EQ.-99) BASEHSV22 = 1  ; Missing data
;;; BASEHSV22-DEFINITION END

;;; BASEMARST-DEFINITION START
IF(MARST.NE.3) BASEMARST = 1  ; Most common
IF(MARST.EQ.3) BASEMARST = ( 1 + THETA(3))
;;; BASEMARST-DEFINITION END

;;; BASEAGE-DEFINITION START
BASEAGE = ( 1 + THETA(4)*(AGE - 24))
;;; BASEAGE-DEFINITION END

;;; BASEA_3MPPSO-DEFINITION START
IF(A_3MPPSO.EQ.1) BASEA_3MPPSO = 1
IF(A_3MPPSO.EQ.-99) BASEA_3MPPSO = 1  ; Missing data
IF(A_3MPPSO.EQ.2) BASEA_3MPPSO = ( 1 + THETA(5))

;;; BASEFIN-DEFINITION START
IF(FIN.EQ.1) BASEFIN = 1  ; Most common YES
IF(FIN.EQ.2) BASEFIN = ( 1 + THETA(6)) ;NO
IF(FIN.EQ.-99) BASEFIN = 1  ; Missing data
;;; BASEHSV22-DEFINITION END
```

```
;;; BASECHILD-DEFINITION START
BASECHILD = ( 1 + THETA(7)*(CHILD - 1))
;;; BASECHILD-DEFINITION END

;;; BASE-RELATION START
BASECOV=BASEHSV22*BASEMARST*BASEAGE*BASEA_3MPPSO*BASEFIN*BASECHILD
;;; BASE-RELATION END

;--------DROPOUT MODEL-----------
A_0(1)=0
TVBASE=THETA(1)
TVBASE = BASECOV*TVBASE
BASE=TVBASE*EXP(ETA(1))

$DES
;--------DROPOUT MODEL-----------
DEL=1E-6
DADT(1)=BASE


$ERROR
;--------DROPOUT MODEL-----------
CHZ = A(1)        ;cumulative hazard
SUR = EXP(-CHZ)           ;survival probability
DEL2=1E-6
HAZNOW=BASE

IF (DV.EQ.0) Y=SUR
IF (DV.EQ.1) Y=SUR*HAZNOW

$THETA
(0, 0.00102)             ; BASE
(-1, 0.937,5)            ; BASEHSV221
(-1, -0.836)             ; BASEMARST1
(-0.1, -0.0764,0.167) ; BASEAGE1
(-1, -0.434,5)           ;BASEA_3MPPSO
(-1, 0.581)              ;BASEFIN
(-1, -0.1)               ;BASCHILD
$OMEGA 0 FIX      ;     BASE

$ESTIMATION METHOD=0 LIKE MAXEVAL=9900 NOABORT PRINT=1 SIGL=9 NSIG=3
MSFO=msfb531_voice
$COV PRINT=E
$TABLE ID TIME DV SUR CHZ FORMAT=s1PE15.8 ONEHEADER NOPRINT FILE=sdtab31_voice
```

NONMEM control stream file for Partners PrEP study

```
;; 1. Based on: run3_3
;; 2. Description: OS-EXP. BASE MODEL. Placebo. STUDY3


$PROBLEM    EXP_MODEL HIV_SURVIVAL PLACEBO

$INPUT ID PLASMA_TFV_LAST=DROP PLASMA_TFV_MED=DROP PLASMA_TFV_LAST_MED=DROP
TISSUE_TFV_LAST=DROP TISSUE_TFV_MED=DROP TISSUE_TFV_LAST_MED=DROP
PBMC_TFVDP_LAST=DROP       PBMC_TFVDP_MED=DROP PBMC_TFVDP_LAST_MED=DROP
FLAG_TFV_TFVDP=DROP TIME PRODUCT HIV_FINAL=DROP  HIV DATE_HIV=DROP TVBI CTBI GCBI
TVBL CTBL GCBL SYP SYPBL PSEXF PSAF OTHP OSEXF OSAF HSV2    HSV2_C LOGVL VL VLDET
VLQUANT OFFDRG=DROP WT HEIGHT=DROP AGE MARST EDU SEX BLCREAT=DROP PREG MINC
PARI CIRC STU=DROP LOGV_LAST VL_LAST VLQ_LAST VLDET_LAST OSEXF_LAST OSAFE_LAST
EVID DV FLAG_SIMUL sittx=DROP count=DROP count2=DROP site=DROP

$DATA        Partners_PD_190424_lastvl.csv
IGNORE (FLAG_SIMUL.EQ.1)
IGNORE (PRODUCT.LT.10)

$SUBROUTINE ADVAN13 TOL=9
$MODEL
COMP (HAZ)

;;time-date (weeks!)
;;dv- hiv
;; Data for estimation FLAG_SIMUL=0
;; Data for simulation FLAG=0 + FLAG=1
;; PRODUCT LT 10= PLACEBO

$PK
;;; BASEAGE-DEFINITION START
IF(AGE.LE.34.00) BASEAGE = ( 1 + THETA(2)*(AGE - 34))
IF(AGE.GT.34.00) BASEAGE = ( 1 + THETA(3)*(AGE - 34))
;;; BASEAGE-DEFINITION END

;;; BASESEX-DEFINITION START
IF(SEX.EQ.0) BASESEX = 1  ; Most common MALE
IF(SEX.EQ.1) BASESEX = ( 1 + THETA(4)) ;FEMALE
;;; BASESEX-DEFINITION END

;;; BASEPARI-DEFINITION START
BASEPARI = ( 1 + THETA(5)*(PARI - 3))
;;; BASEPARI-DEFINITION END

;;; BASE-RELATION START
BASECOV=BASEAGE*BASESEX*BASEPARI
;;; BASE-RELATION END

A_0(1)=0
TVBASE=THETA(1)
TVBASE = BASECOV*TVBASE
BASE=TVBASE*EXP(ETA(1))

$DES
;--------DROPOUT MODEL-----------
DEL=1E-6
```

```
DADT(1)=BASE
$ERROR
;--------DROPOUT MODEL-----------
CHZ = A(1)                    ;cumulative hazard
SUR = EXP(-CHZ)               ;survival probability
DEL2=1E-6
HAZNOW=BASE

IF (DV.EQ.0) Y=SUR
IF (DV.EQ.1) Y=SUR*HAZNOW

$THETA
(0, 0.00016)                  ; BASE
(-100000, -0.31,0.059)   ; BASEAGE1
(-0.033, 0.0314,100000) ; BASEAGE2
(-1, 0.651,5)                 ; BASesex
(-1,-0.05)                    ; BASEPARI1

$OMEGA 0 FIX  ;      BASE

$ESTIMATION METHOD=0 LIKE MAXEVAL=9900 NOABORT PRINT=1 SIGL=9 NSIG=3 MSFO=msfb54_4
$COV PRINT=E
$TABLE ID TIME DV SUR CHZ FORMAT=s1PE15.8 ONEHEADER NOPRINT FILE=sdtab4_4
```

NONMEM control stream file for BTS study

```
;; 1. Based on: run23
;; 2. Description: SEX drgFreq OS-EXP Placebo. STUDY4

$PROBLEM    EXP_MODEL HIV_SURVIVAL PLACEBO

$INPUT ID STD=DROP STPD=DROP SIT=DROP PRODUCT SEX EDY AGE BLC=DROP BLCRCL=DROP
WT=DROP DG3M=DROP DGFR HET3=DROP HETS=DROP LIV=DROP PART=DROP PARTC=DROP
CASSE=DROP CASCON=DROP ANA3=DROP ANAF=DROP ANAF2=DROP MSM=DROP RECE=DROP
ANC=DROP HIVPD=DROP POSAT=DROP HIV_FIN HIV_LA=DROP ADH=DROP PTD=DROP
PBTD=DROP PKD=DROP PLASM1=DROP PLASM2=DROP PLASM3=DROP PBMC1=DROP PBMC2=DROP
PBMC3=DROP TIS1=DROP TIS2=DROP TIS3=DROP WK=DROP TIME DV EVID FLAG_SIMUL


$DATA    placebo_bangkok_NM.csv
IGNORE (FLAG_SIMUL.EQ.1)

$SUBROUTINE ADVAN13 TOL=9
$MODEL
COMP (HAZ)

;;time-date (weeks!)
;;dv- hiv
;; Data for estimation FLAG_SIMUL=0
;; Data for simulation FLAG=0 + FLAG=1

$PK
;;; BASESEX-DEFINITION START
IF(SEX.EQ.2) BASESEX = 1  ; Most common MALE
IF(SEX.EQ.1) BASESEX = ( 1 + THETA(2)) ;FEMALE
;;; BASESEX-DEFINITION END

;;; BASEDGFR-DEFINITION START
IF(DGFR.EQ.-99) BASEDGFR = 1  ; Missing data
IF(DGFR.EQ.2) BASEDGFR = 1  ; Most common
IF(DGFR.EQ.1) BASEDGFR = 1
IF(DGFR.EQ.3) BASEDGFR = ( 1 + THETA(3)) ;DRUG INJECTION DAIly
;;; BASEDGFR-DEFINITION END

;;; BASE-RELATION START
BASECOV=BASESEX*BASEDGFR
;;; BASE-RELATION END

;--------DROPOUT MODEL-----------
A_0(1)=0
TVBASE=THETA(1)
TVBASE = BASECOV*TVBASE
BASE=TVBASE*EXP(ETA(1))

$DES
;--------DROPOUT MODEL-----------
DEL=1E-6
DADT(1)=BASE

$ERROR
;--------DROPOUT MODEL-----------
CHZ = A(1)        ;cumulative hazard
```

```
SUR = EXP(-CHZ)           ;survival probability
DEL2=1E-6
HAZNOW=BASE

IF (DV.EQ.0) Y=SUR
IF (DV.EQ.1) Y=SUR*HAZNOW

$THETA
(0, 0.00064)       ; BASE
(-1, 1.76,5)        ; BASESEX1
 (-1,1)             ; BASEDGFR2
$OMEGA 0 FIX ;     BASE

$ESTIMATION METHOD=0 LIKE MAXEVAL=9900 NOABORT PRINT=1 SIGL=9 NSIG=3 MSFO=msfb525
$COV PRINT=E
$TABLE ID TIME DV SUR CHZ FORMAT=s1PE15.8 ONEHEADER NOPRINT FILE=sdtab25
```

<u>NONMEM control stream file for TDF2 study</u>

```
;; 2. Description: OS-EXP. BASE MODEL. Placebo. STUDY5

$PROBLEM    EXP_MODEL HIV_SURVIVAL PLACEBO

$INPUT ID SIT RACE AGE MAR EDU NPAR NVAG NCOND GON CLAM SYP TRI WGT BLCRT CRCL
SEX STDA=DROP HIVP=DROP HIVFIN PSDA=DROP STDA=DROP GONN=DROP GONND=DROP
CLAMM=DROP CLAMMD=DROP SYPP=DROP SYPPD=DROP TRII=DROP TRIID=DROP PIAD=DROP
PRODUCT PKD=DROP PTFVL=DROP PTFVM=DROP PTFVLM=DROP PTFVL=DROP PTFVM=DROP
PTFVLM=DROP TTFVL=DROP TTFVM=DROP TTFVLM=DROP WTE=DROP DV TIME EVID
FLAG_SIMUL

$DATA       placebo_tdf2_NM.csv
IGNORE (FLAG_SIMUL.EQ.1)

$SUBROUTINE ADVAN13 TOL=9
$MODEL
COMP (HAZ)

;;time-date (weeks!)
;;dv- hiv
;; Data for estimation FLAG_SIMUL=0
;; Data for simulation FLAG=0 + FLAG=1

$PK

;--------DROPOUT MODEL-----------
A_0(1)=0
BASE=THETA(1)*EXP(ETA(1))

$DES
;--------DROPOUT MODEL-----------
DEL=1E-6
DADT(1)=BASE

$ERROR
;--------DROPOUT MODEL-----------
CHZ = A(1)        ;cumulative hazard
SUR = EXP(-CHZ)          ;survival probability
DEL2=1E-6
HAZNOW=BASE

IF (DV.EQ.0) Y=SUR
IF (DV.EQ.1) Y=SUR*HAZNOW

$THETA
(0, 0.00068) ; BASE

$OMEGA 0 FIX  ;      BASE

$ESTIMATION METHOD=0 LIKE MAXEVAL=9900 NOABORT PRINT=1 SIGL=9 NSIG=3
MSFO=msfb51_td
$COV PRINT=E
$TABLE ID TIME DV SUR CHZ FORMAT=s1PE15.8 ONEHEADER NOPRINT FILE=sdtab1_td
```
